# Epidemiological characteristics and molecular evolution mechanisms of carbapenem-resistant hypervirulent *Klebsiella pneumoniae*

**DOI:** 10.3389/fmicb.2022.1003783

**Published:** 2022-09-12

**Authors:** Yu-Ling Han, Xu-Hui Wen, Wen Zhao, Xi-Shan Cao, Jian-Xun Wen, Jun-Rui Wang, Zhi-De Hu, Wen-Qi Zheng

**Affiliations:** ^1^Department of Laboratory Medicine, The Affiliated Hospital of Inner Mongolia Medical University, Hohhot, China; ^2^Department of Parasitology, The Basic Medical College of Inner Mongolia Medical University, Hohhot, China; ^3^Department of Medical Experiment Center, The Basic Medical Sciences College of Inner Mongolia Medical University, Hohhot, China

**Keywords:** *Klebsiella pneumoniae*, carbapenem-resistant, hypervirulent, hybrid plasmid, evolution

## Abstract

Carbapenem-resistant hypervirulent *Klebsiella pneumoniae* (CR-hvKP), a type of *Klebsiella pneumoniae* (KP) that exhibits hypervirulence and carbapenem resistance phenotypes, can cause severe infections, both hospital- and community-acquired infections. CR-hvKP has brought great challenges to global public health and is associated with significant morbidity and mortality. There are many mechanisms responsible for the evolution of the hypervirulence and carbapenem resistance phenotypes, such as the horizontal transfer of the plasmid carrying the carbapenem resistance gene to hypervirulent *Klebsiella pneumoniae* (hvKP) or carbapenemase-producing *Klebsiella pneumoniae* (CRKP) acquiring a hypervirulence plasmid carrying a virulence-encoding gene. Notably, KP can evolve into CR-hvKP by acquiring a hybrid plasmid carrying both the carbapenem resistance and hypervirulence genes. In this review, we summarize the evolutionary mechanisms of resistance and plasmid-borne virulence as well as the prevalence of CR-hvKP.

## Introduction

**Figure 1:**
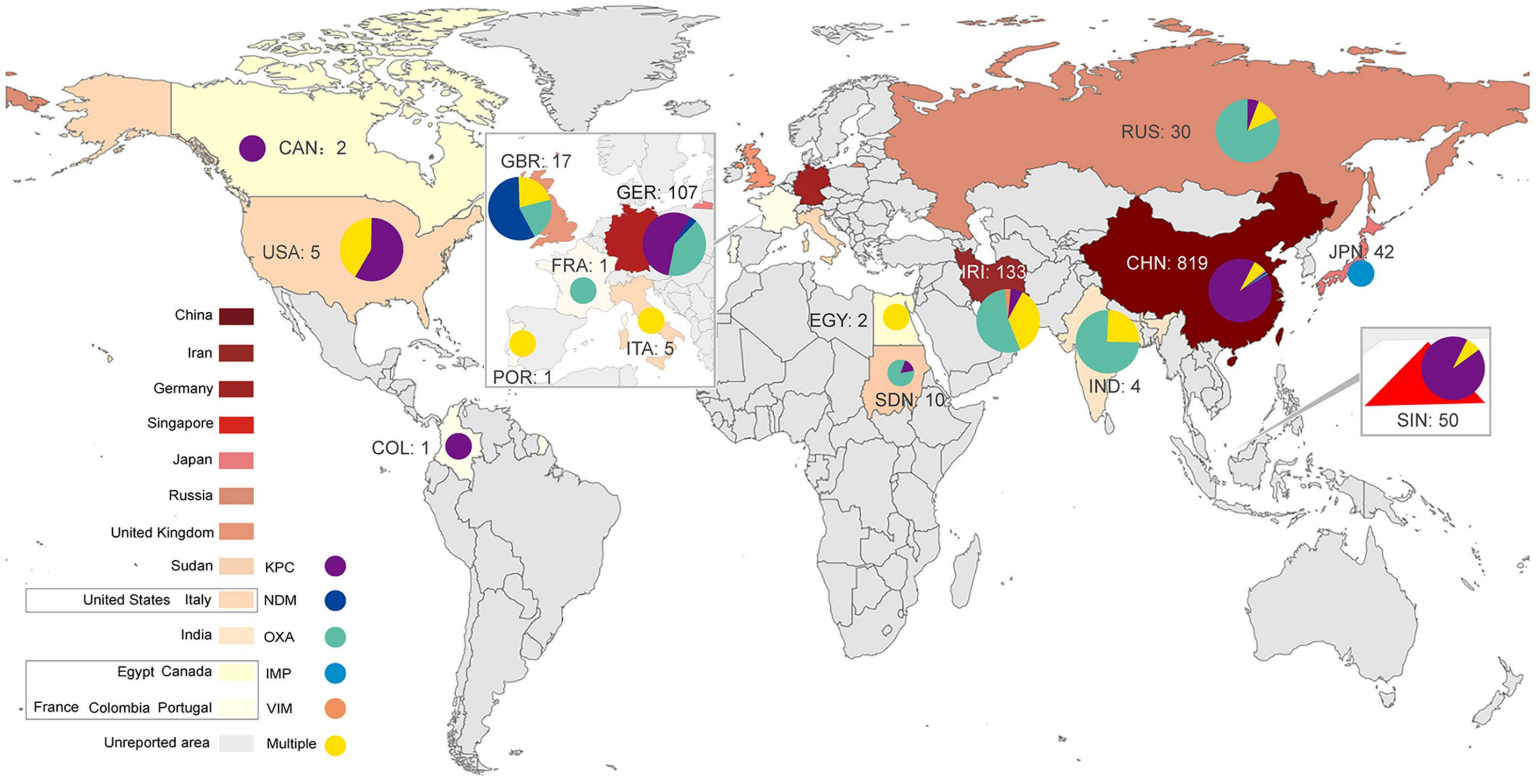
Global distribution of CR-hvKP (2015–2022). KPC, *Klebsiella pneumoniae* carbapenemase; NDM, New Delhi metallo-β-lactamase; VIM, Verona integron-encoded metallo-β-lactamase; IMP, imipenemase; OXA, oxacillinase.

*Klebsiella pneumoniae* (KP) is an opportunistic pathogen that can cause infectious diseases in the urinary tract, respiratory tract, blood and soft tissue (Podschun and Ullmann, 1998). KP can be categorized into classic *Klebsiella pneumoniae* (cKP) and hypervirulent *Klebsiella pneumoniae* (hvKP) according to its phenotypic and genotypic characteristics. HvKP has higher virulence than cKP (Shon et al., 2013) and can cause severe infectious diseases, especially pyogenic liver abscess (Wang et al., 1998), endophthalmitis (Liu et al., 1986), and meningitis (Xu et al., 2019). It was first identified in seven patients in 1986 (Liu et al., 1986). These patients suffered from severe liver abscesses and endophthalmitis. Despite antibiotic therapy being initiated in a timely manner, six patients lost vision, and one was visually impaired (Liu et al., 1986). To date, many investigations have reported the prevalence of hvKP (Shon et al., 2013). In recent years, partially due to antibiotic abuse, the prevalence of carbapenem-resistant *Klebsiella pneumoniae* (CRKP) has increased (Doi and Paterson, 2015; Chung, 2016). HvKP and CRKP can evolve into carbapenem-resistant hypervirulent *Klebsiella pneumoniae* (CR-hvKP) by acquiring the plasmids carrying the carbapenem resistance gene or virulence-encoding gene, respectively. Notably, cKP can evolve into CR-hvKP by acquiring a hybrid plasmid carrying both the carbapenem resistance and hypervirulence genes (He et al., 2022). Therefore, CR-hvKP exhibits both hypervirulence and carbapenem resistance phenotypes, which may cause severe infections in individuals that are difficult to treat with current antibiotics, should attracted worldwide attention (Gu et al., 2018a). At present, CR-hvKP has spread worldwide (Figure 1) and poses a great threat to human public health. In this study, we summarize the resistance mechanisms and virulence evolution mechanism of CR-hvKP.

## Mechanism of carbapenem resistance in CR-hvKP

There are three mechanisms responsible for the evolution of CR-hvKP, including carbapenemase production, activation of the efflux pump system and loss of outer membrane proteins (OMPs) expression (Liao et al., 2020). These mechanisms are briefly introduced in this review.

### Carbapenemase production

Multidrug resistance genes in plasmids and genomes regulate the resistance characteristics of KP. These genes can encode aminoglycosides, extended-spectrum β-lactamase (ESBL), AmpC β-lactamases or carbapenemases (Tzouvelekis et al., 2012). Carbapenemase production is an essential mechanism of carbapenem resistance in CRKP (Bonomo et al., 2018). According to the Ambler classification, carbapenemases can be categorized into three classes: A, B and D (Queenan and Bush, 2007). Carbapenemase class A can hydrolyze nearly all β-lactam antibiotics (Rasmussen and Bush, 1997). *Klebsiella pneumoniae* carbapenemases (KPCs) are the primary class A enzymes abundant in CRKP (Brink, 2019). This enzyme was first identified in 1996 in the United States from a KP isolate (Chen et al., 2014). There are many subtypes of KPCs, including KPC-2 to KPC-13 (Tzouvelekis et al., 2012). Notably, the KPC genes, *bla*_*KPC*_, in the genome and plasmid share transmission features, such as horizontal transfer or cloning to other strains mediated by genetic elements (Rasmussen and Bush, 1997; Chen et al., 2014; Chew et al., 2017). When hvKP obtains *bla_KPC_*, it can evolve to CR-hvKP with both high virulence and carbapenem-resistance characteristics. Globally, many *bla_KPC_*-positive CR-hvKP strains have been reported since 2010 (Lan et al., 2021). A report showed that the most common subtypes of *bla_KPC_* in the USA are *bla_KPC-2_* and *bla_KPC-3_*. In Asia (especially China) and Europe, the most frequent type is *bla_KPC-2_* (Yang et al., 2021a). The global prevalence of the *bla_KPC_*-positive CR-hvKP strain is summarized in Figure 1; Table 1.

**Table 1:** Global geographic distribution of isolates obtained carbapenem resistance gene from June 2015 to April 2022.

**Country**	***bla***_***KPC***_	***bla***_***NDM***_	***bla***_***OXA***_	***bla***_***IMP***_	***bla***_***VIM***_	**Multiple carbapenem genes**	**References**
China	87.3% (715/819)	3.3% (27/819)	1.3% (11/819)	0.3% (3/819)	0.2% (2/819)	7.6% (61/819) *bla_KPC_*, *bla_NDM_*, *bla_OXA_*, *bla_VIM_* (8/61); *bla_KPC_*, *bla_OXA_*, *bla_VIM_* (2/61); *bla_NDM_*, *bla_OXA_*, *bla_VIM_* (1/61); *bla_KPC_*, *bla_NDM_* (10/61); *bla_KPC_*, *bla_OXA_* (35/61); *bla_KPC_*, *bla_IMP_* (1/61); *bla_KPC_*, *bla_VIM_* (1/61); *bla_NDM_*, *bla_OXA_* (2/61); *bla_NDM_*, *bla_VIM_* (1/61)	Yao et al., 2015; Zhang et al., 2015; Liu et al., 2016; Zhang et al., 2016; Liu et al., 2017; Zhan et al., 2017; Dong et al., 2018; Feng et al., 2018; Huang et al., 2018; Wei et al., 2018; Gu et al., 2018a,b; Dong et al., 2019; Liu et al., 2019; Liu and Su, 2019; Shen et al., 2019; Shu et al., 2019; Xu et al., 2019; Yuan et al., 2019; Zhou et al., 2019; Li et al., 2019b; Hao et al., 2020; Jin et al., 2020; Liu et al., 2020; Yang et al., 2020; Zhang et al., 2020; Zheng et al., 2020; Zhou et al., 2020; Li et al., 2020a,b; Chen et al., 2021; Huang et al., 2021; Jin et al., 2021; Liu et al., 2021; Shao et al., 2021; Su et al., 2021; Xie et al., 2021; Yan et al., 2021; Zhang et al., 2021; Zhao et al., 2021; Zhou et al., 2021; Li et al., 2021a,b; Yang et al., 2021b; Wei et al., 2022; Zhou et al., 2022; Yang et al., 2022b
India	0% (0/4)	0% (0/4)	75.0% (3/4)	0% (0/4)	0% (0/4)	25.0% (1/4) *bla_NDM_*, *bla_OXA_* (1/1)	Shankar et al., 2016; Mukherjee et al., 2020
Iran	0% (0/133)	7.6% (10/133)	54.9% (73/133)	0% (0/133)	3.7% (5/133)	33.8% (45/133) *bla_NDM_*, *bla_OXA_* (41/41)	Mohammad Ali Tabrizi et al., 2018; Pajand et al., 2020; Solgi et al., 2020; Banerjee et al., 2021; Bolourchi et al., 2021; Sanikhani et al., 2021
Singapore	90.0% (45/50)	0% (0/50)	0% (0/50)	0% (0/50)	0% (0/50)	10.0% (5/50) *bla_KPC_*, *bla_OXA_* (5/5)	Octavia et al., 2019; Chen et al., 2020
Japan	0% (0/42)	0% (0/42)	0% (0/42)	100.0% (42/42)	0% (0/42)	0% (0/42)	Harada et al., 2019; Yonekawa et al., 2020
Russia	0% (0/30)	0.7% (2/30)	70.0% (21/30)	0% (0/30)	0% (0/30)	23.3% (7/30) *bla_NDM_*, *bla_OXA_* (7/7)	Lev et al., 2018; Shaidullina et al., 2020; Starkova et al., 2021
Italy	0% (0/5)	0% (0/5)	0% (0/5)	0% (0/5)	0% (0/5)	100% (5/5) *bla_NDM_*, *bla_OXA_* (5/5)	Scaltriti et al., 2020; Lorenzin et al., 2022
United Kingdom	0% (0/17)	58.9% (10/17)	23.5% (4/17)	0% (0/17)	0% (0/17)	17.6% (3/17) *bla_NDM_*, *bla_OXA_* (3/3)	Roulston et al., 2018; Turton et al., 2018
France	0% (0/1)	0% (0/1)	100.0% (1/1)	0% (0/1)	0% (0/1)	0% (0/1)	Beyrouthy et al., 2020
Germany	56.0% (60/107)	4.7% (5/107)	39.3% (42/107)	0% (0/107)	0% (0/107)	0% (0/107)	Becker et al., 2018
Portugal	0% (0/1)	0% (0/1)	0% (0/1)	0% (0/1)	0% (0/1)	100.0% (1/1) *bla_KPC_*, *bla_OXA_* (1/1)	Mendes et al., 2022
United States	60.0% (3/5)	0.0% (0/5)	0.0% (0/5)	0.0% (0/5)	0.0% (0/5)	40.0% (2/5)` *bla_KPC_*, *bla_OXA_* (2/2)	Krapp et al., 2017; Karlsson et al., 2019
Canada	100% (2/2)	0% (0/2)	0% (0/2)	0% (0/2)	0% (0/2)	0% (0/2)	Mataseje et al., 2019
Colombia	100% (1/1)	0% (0/1)	0% (0/1)	0% (0/1)	0% (0/1)	0% (0/1)	Saavedra et al., 2021
Egypt	0% (0/2)	0% (0/2)	0% (0/2)	0% (0/2)	0% (0/2)	100.0% (2/2) *bla_KPC_*, *bla_NDM_* (1/2); *bla_KPC_*, *bla_OXA_* (1/2)	Ahmed et al., 2021; Yang et al., 2022c
Sudan	0% (0/10)	20% (2/10)	80% (8/10)	0% (0/10)	0% (0/10)	0% (0/10)	Albasha et al., 2020
Total	67.2% (826/1229)	4.6% (56/1229)	13.2% (163/1229)	3.7% (45/1229)	0.6% (7/1229)	10.7% (132/1229)	

Class B carbapenemase enzymes, also named metallo-β-lactamases (MBLs), mainly include New Delhi metallo-β-lactamase (NDM-1), Verona integron-encoded metallo-β-lactamase (VIM-1) and imipenemase (IMP). These enzymes are characterized by one or two zinc ions as active centers (Tzouvelekis et al., 2012; Bonomo et al., 2018). MBLs can catalyze the hydrolysis of almost all β-lactam antibiotics except for monobactams (Jeon et al., 2015). Class D enzymes mainly include oxacillinase 48 (OXA-48) and 181 (OXA-181). These enzymes have carbapenemase activity, but their carbapenem hydrolysis activity is weak. Notably, the transmission of class B and class D enzyme genes depends mainly on plasmids (Tzouvelekis et al., 2012; Bonomo et al., 2018), making their distribution more regional. Class B (e.g., NDM-1, VIM-1) and D (e.g., OXA-48, OXA-181) enzymes are primarily distributed in Asia and Europe (Nordmann et al., 2012). In Asian countries such as India and Iran and in European countries such as Russia and Italy, the majority of CR-hvKP isolates were OXA-48-positive strains, followed by NDM-1- and VIM-1-positive strains (Figure 1; Supplementary Table S1). In 2018, an investigation from Iran reported five patients infected with rare strains of K1 and ST23 CR-hvKP, which carry the *bla_VIM-2_* gene (Mohammad Ali Tabrizi et al., 2018). Notably, four of these patients died during hospitalization (Mohammad Ali Tabrizi et al., 2018), indicating the hypervirulence of this type of CR-hvKP. In addition, some Asian countries, such as China and Japan, have occasionally reported IMP-positive CR-hvKP strains (Zhang et al., 2015; Harada et al., 2019).

In conclusion, the distribution of carbapenemases differs according to geographical features. CR-hvKP strains that produce class A enzymes are highly prevalent in Asia and America, and a handful of strains produce class B and D enzymes in Europe (Figure 1; Supplementary Table S1). However, the increase in factors such as global immigration may lead to changes in the linkages between these bacterial resistance mechanisms and regions or cities (Bonomo et al., 2018). Therefore, the areas with low prevalence cannot be ignored when detecting CR-hvKP-related resistance genes.

Furthermore, CR-hvKP strains simultaneously producing two or more carbapenemases can cause serious infectious diseases with high mortality. These types of CR-hvKP have been reported in many countries and regions (Figure 1; Supplementary Table S1). For example, CR-hvKP strains producing NDM and OXA-48 were reported in Italy and Iran in 2020 (Scaltriti et al., 2020; Solgi et al., 2020; Bolourchi et al., 2021). A CR-hvKP strain carrying *bla_NDM-1_*, *bla_NDM-5_* and *bla_OXA-48_* carbapenem resistance genes was reported in northern Italy in 2022 (Lorenzin et al., 2022). In particular, a CR-hvKP strain carrying *bla_KPC-2_* and *bla_OXA-48_* genes was reported in Egypt in the same year (Yang et al., 2022c). This CR-hvKP isolate contained two resistance plasmids: pEBSI041-2 (a *bla_OXA-48_* gene carrier) and pEBSI041-3 (a *bla_KPC-2_* gene carrier). pEBSI041-3 can be transferred with the assistance of plasmid pEBSI041-2, and the two plasmids can fuse into a larger plasmid carrying both *bla_OXA-48_* and *bla_KPC-2_* genes during co-transfer. Plasmids carrying different carbapenem resistance genes could be transferred through gene recombination, rearrangement and the formation of fusion plasmids, resulting in more complex resistance mechanisms in CR-hvKP strains (Yang et al., 2022c).

### Activation of the efflux pump system and decreased expression or loss of outer membrane porins

**Figure 2:**
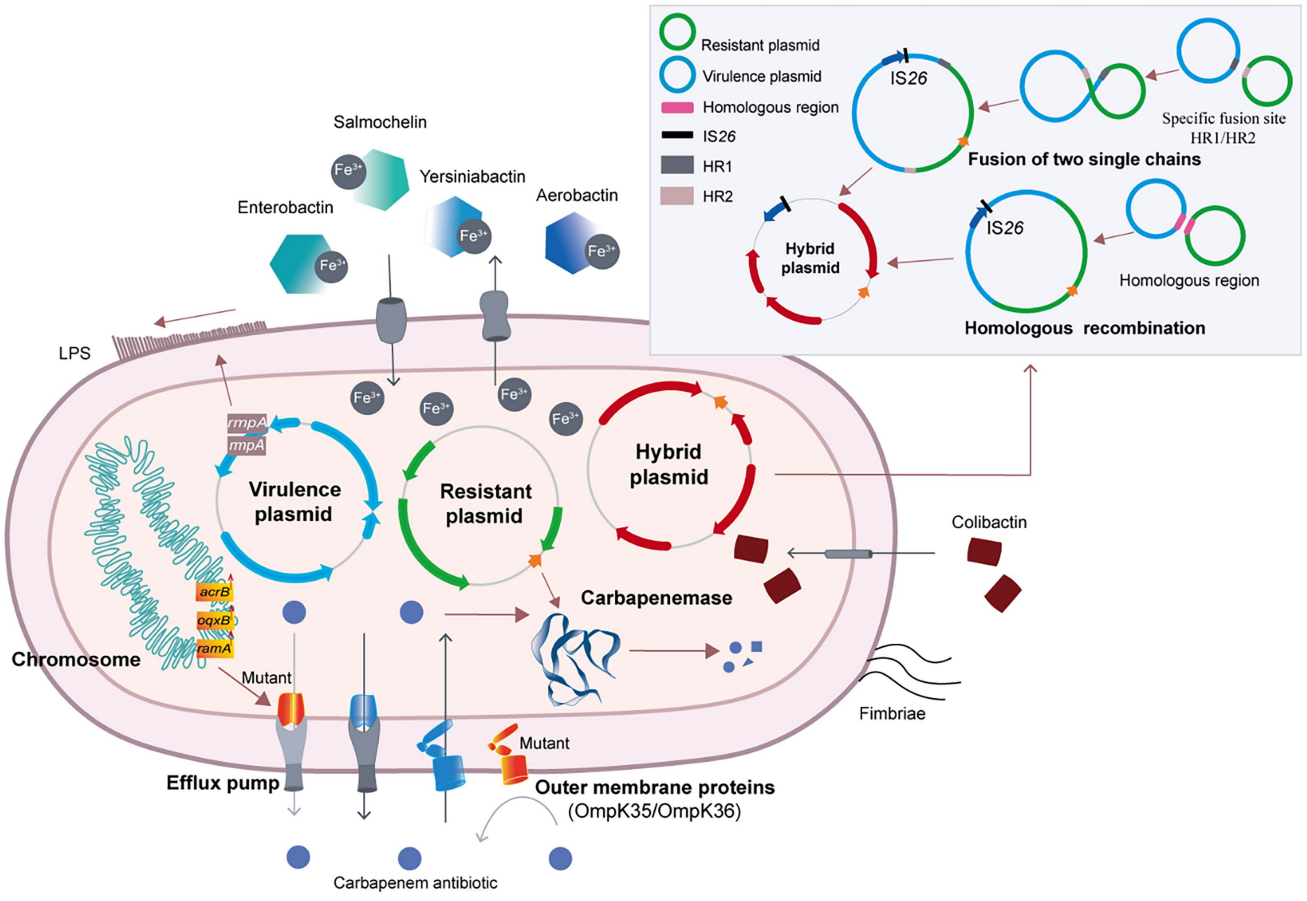
The molecular evolution and carbapenem-resistance mechanism of CR-hvKP.

KP can rapidly pump intracellular antibiotics out of the cell through the efflux pump system, thus decreasing the concentration of antibiotics in the bacteria. Activation of the efflux pump system and decreased expression of outer membrane porins (OMPs) are two common mechanisms of carbapenem resistance in CRKP (Figure 2). The tripartite efflux system AcrAB-TolC is the archetypal resistance-nodulation-cell division (RND) efflux pump and contributes to multidrug resistance (Tam et al., 2021). In KP, the AcrAB-TolC efflux pump can be regulated by RamA, an AraC/XylS transcriptional activator (Xu et al., 2021a). Upregulation of *ramA* enhances the expression of *acrAB* and *tolC*, resulting in increased translation of the AcrAB-TolC pump proteins, ultimately leading to multidrug resistance (Nishino and Yamaguchi, 2004). In 2021, 17 CRKP strains that cause urinary tract infections were isolated. The carbapenem resistance mechanism of 11 KPC and/or VIM-positive CRKP strains was mainly related to the overexpression of the *ramA* gene and upregulation of the *acrB* and *oqxB* genes. Only 6 strains exhibited other resistance mechanisms (Lee et al., 2021). Therefore, overexpression of efflux pumps is one of the major resistance mechanisms of CRKP (Du et al., 2018).

Moreover, mutation of the efflux pump is another mechanism for changing the activity of the efflux pump (Figure 2). In 2021, researchers found that the transcription factor *ramA* could downregulate the efflux pump genes *acrB* and *oqxB* after mutation, resulting in a significant decrease in the MIC of tigecycline in carbapenem-susceptible cKP (Xu et al., 2021a). When the efflux pump regulator is mutated, the corresponding antibiotic resistance may also reduce bacterial adaptability and virulence (Du et al., 2018).

OMPs are the channels through which antibiotics enter bacteria. Similar to efflux pumps, changes in OMPs also affect the resistance phenotype of bacteria (Li et al., 2015). OmpC, OmpD, OmpE, OmpF, and PhoE are common outer membrane pore proteins in clinical *Enterobacter* strains (Vergalli et al., 2020). OmpK36 belonging to the OmpC family and OmpK35 belonging to the OmpF family are common in KP (Pagès et al., 2008). In CRKP, changes in the expression levels of OmpK35 and OmpK36 can have important effects. Low expression of OmpK35 is one of the important mechanisms for the antibiotic resistance of ESBL-producing CRKP (Wang et al., 2009). OmpK36 can reduce the sensitivity of ESBL and AmpC β-lactamase of CRKP and is closely related to the resistance of CRKP to carbapenems (Wang et al., 2009). In a retrospective study with 28 cases of CRKP infections from 9 cities in China, 5 CR-hvKP strains were identified (Zhang et al., 2015). The expression levels of OmpK35 and OmpK36 were decreased in 2 CR-hvKP strains (Zhang et al., 2015), indicating that the low expression levels of OmpK35 and OmpK36 are potential causes of resistance to carbapenems (Zhang et al., 2015).

In addition, in 2009, a study with 28 CRKP strains found that 9 CRKP isolates with high levels of ertapenem resistance consistently lacked both OmpK35 and OmpK36 porins (Doumith et al., 2009). Sequencing of the *ompK35* and *ompK36* genes revealed diverse types of disruption in most isolates, including point mutations or the presence of insertion sequences (Iss), such as IS*1* and IS*10* (Doumith et al., 2009), which indicated that the carbapenem resistance characteristics of the CRKP strains are likely to be influenced by these mutations in OMPs (Doumith et al., 2009).

## Virulence evolution mechanism of CR-hvKP

### HvKP and virulence factors

#### Hypermucoviscosity

The outermost capsule polysaccharides are key for the hypermucoviscous phenotype of hvKP. The capsular polysaccharide can protect bacteria from harmful environmental factors, such as antimicrobial compounds, and improve their virulence, so hvKP is traditionally thought to have hypermucoviscosity characteristics (Russo and Marr, 2019). Therefore, the string test to detect viscosity is a classic method to identify hvKP. Briefly, the monoclonal colonies on the agar plate are gently picked up with a bacteriological loop, and a length of viscous filament >5 mm is judged as a positive result in the string test (Lan et al., 2021).

Many studies have shown that two capsule regulator genes, mucoid phenotype A (*rmpA*), and mucoid phenotype A2 (*rmpA2*), on the virulence plasmid are hvKP-specific virulence factors (Chen and Chen, 2021). Overexpression of the *rmpA* gene increases the virulence of KP in a mouse infection model (Lin et al., 2020), which indicates that the highly virulent phenotype is associated with high expression of the *rmpA* gene (Lin et al., 2020). However, some studies have indicated that there is no correlation between hypermucoviscosity and virulence. A retrospective study that included 28 CRKP isolates collected from 9 regions in China showed that although 2 strains were positive in the string test, they did not carry the *rmpA* gene (Zhang et al., 2015). In addition, 5 CRKP strains carried the *rmpA* gene but were negative in the string test (Zhang et al., 2015). Therefore, the string test (hypermucoviscosity) is not a sensitive and specific method to identify hvKP. Consequently, the identification of hvKP should include the identification of invasion-related clinical characteristics and other virulence-associated determinants.

#### Capsule serotype

Variations in the capsular polysaccharide composition result in different capsules (Choby et al., 2020). The K-antigens encoded by the *cps* (capsule polysaccharide synthesis) locus belong to the bacterial capsule polysaccharides (CPS) with genetic diversity. It has been reported that there are nine kinds of *cps* operons (Chung The et al., 2015). Among them, the *wzi* gene is one of the most important determinants of CPS, and usually, KP serotypes can be identified by detecting the *wzi* gene (Wyres et al., 2016; Choby et al., 2020).

Recent studies have shown that K-antigens are associated with hvKP infection (Follador et al., 2016). The number of serotypes has been estimated to be 77 for K-antigens (Choby et al., 2020). K1 and K2 are the most common capsule serotypes and the major pathogen capsule serotypes causing invasive infectious diseases (Fung et al., 2002; Chuang et al., 2006; Su et al., 2020). Therefore, capsule serotypes K1 and K2 are typical characteristics of hvKP (Choby et al., 2020). Other capsule serotypes, such as K20 (Zhan et al., 2017), K47 (Huang et al., 2018), K54 (Yuan et al., 2019), K57 (Zhang et al., 2020b) and K64 (Liu et al., 2017; Zhang et al., 2020b), can also cause serious infectious diseases and have strong virulence. Thus, capsule type may be associated with bacterial virulence or can enhance virulence (Russo and Marr, 2019; Choby et al., 2020), but it is not the only virulence-associated determinant of hvKP.

A study with 85 hvKP and 90 cKP strains assessed the diagnostic accuracy rate of K1, K2, K5, K20, K54, and K57 for hvKP (Russo et al., 2018). Although the diagnostic specificity of these capsule serotypes was above 90%, the sensitivity of K1 and K2 of hvKP was only 55 and 20%, respectively. For K5, K20, K54, and K57, the sensitivity was less than 8%. However, it is worth noting that, for the combination of the K1, K2, K5, K20, K54, and K57 capsule types, the sensitivity and specificity were 93 and 88%, respectively, and the accuracy rate was 90% (Russo et al., 2018). Therefore, combining multiple capsule types in clinical treatment can also be used to accurately diagnose hvKP.

#### Siderophores

HvKP strains can release siderophores (SPs) when they invade the host body. SPs can acquire iron from the binding protein in the host body and then bind to the SP-specific receptor to re-enter KP cells. Eventually, hvKP used the “steal” iron to grow (Russo et al., 2014; Russo and Marr, 2019; Choby et al., 2020). There are four SPs secreted by hvKP, namely enterobactin, salmochelin, yersiniabactin and aerobactin. Site-specific gene disruptions were performed on the four SPs in previous research. Only when the aerobactin gene *iucA* was destroyed was the *ex vivo* growth/survival of hvKP in human ascites fluid and serum significantly decreased, and the virulence of hvKP in the mouse infection model was also significantly decreased. In contrast, the other three iron carriers did not show the features mentioned above (Russo et al., 2014, 2015). These results indicate that aerobactin in SPs is the primary determinant of the virulence of hvKP. In addition, a diagnostic accuracy study suggested that the expression levels of *peg-344*, *iroB* and *iucA* can accurately predict the virulence of hvKP (Russo et al., 2018). *Peg-344*, *iroB* and *iucA* have demonstrated >0.95 diagnostic accuracy in identifying hvKP strains. Therefore, SP-related encoding genes, especially *iucA*, are key markers in the identification of hvKP (Russo et al., 2018).

#### Virulence plasmids

As mentioned above, cKP can transform into hvKP with a hypervirulence virulence phenotype by acquiring virulence plasmids. These virulence plasmids usually carry many virulence coding genes, such as hypermucoviscous phenotype genes (*rmpA* and *rmpA2*), SP-related genes (*iucABCD-iutA, iroBCDN, ybtAEPQTUX* and *entABCDEFS*) and genes encoding tellurite and silver resistance (*terABCDEWXZ*, *silCERS*). Among them, *iroB*, *iucA*, *peg-344*, *p-rmpA* and *p-rmpA2* are the most accurate and characteristic molecular markers for defining hvKP on virulence plasmids (Hsu et al., 2011; Bulger et al., 2017; Russo et al., 2018). To date, the 224 kb plasmid pNTUH-K2044 on K1-ST23 KP strain NTUH-K2044 and its highly similar plasmid pLVPK on K2-ST86 KP strain CG43 are the most reported virulence plasmids (Chen et al., 2004; Wu et al., 2009). The virulence of KP strains carrying these virulence plasmids (pNTUH-K2044, pLVPK or pLVPK-like virulence plasmid) can be significantly increased (Gu et al., 2018a). In a study, the hvKP strain K2602 carrying the virulence plasmid pK2602 was used as the donor, and the ST11 CRKP strain HS11286 and *E. coli* J53 were used as the recipient strains for conjugation experiments. It was found that the transconjugants (HS11286-K2606 and J53-K2606) acquired the virulence plasmid pK2606, which can encode aerobactin from K2602, and exhibited increased siderophore production, though the result for HS11286-K2606 was more significant. These two transconjugants could also cause high mortality in *Galleria mellonella* and mice. Therefore, virulence plasmids are an important cause of the hypervirulence phenotype (Tian et al., 2021). More importantly, many studies have reported fusion plasmids integrating both carbapenem resistance and hypervirulence phenotypes in recent years (Lorenzin et al., 2022; Zhou et al., 2022; Yang et al., 2022b), which signifies that hvKP has evolved into a “superbug.”

### CRKP

Bloodstream infection (BSI) and pneumonia caused by CRKP can have higher mortality than CRKP urinary colonization (Hauck et al., 2016). Some scholars have also carried out relevant studies on CRKP virulence. In 2015, researchers infected mice with KPC-producing ST11 CRKP and ST258 CRKP strains and compared their virulence. The results showed that the virulence of these two strains was low, indicating that KPC production is irrelevant to the virulence of these strains (Chiang et al., 2016). Similarly, in 2017, researchers in the United States isolated 4 CRKP strains from patients with necrotizing skin and soft tissue infections (Krapp et al., 2017). All 4 CRKP strains presented low virulence in the mouse acute pneumonia model. However, in a mouse subcutaneous tissue infection model, one CRKP strain with a positive string test result caused severe skin abscess and finally spread to the liver (Krapp et al., 2017). These results indicate that the virulence level of the CRKP strain with a positive string test result might be tissue-dependent (Krapp et al., 2017). Moreover, a study that included 56 KPC-producing CRKP strains isolated from hospitalized patients in China in 2018 compared the virulence characteristics of different sequence types. Among the CRKP stains, 43 were ST11, 6 were ST147, 4 were ST15, and 3 were ST1456, ST65, and ST23. According to the *Galleria mellonella* infection model, the virulence of the ST11 CRKP strains was lower than that of other sequence types (Liu et al., 2017). This result indicated that the virulence of CRKPs with different sequence types varies. The virulence characteristic of the ST11 CRKP strain, the most common in Asia, was low before acquisition of the virulence-associated determinants.

### CR-hvKP

The complex and diverse evolutionary mechanisms are the key factors that can improve the virulence and antibiotic resistance of KP stains and increase the relevant morbidity and mortality of the associated infectious diseases. This is the primary reason why the low-virulence cKP and CRKP strains, or the strains with low antibiotic resistance, evolved to “superbug” CR-hvKP with the characteristic of stronger virulence, high carbapenem resistance and global transmission. The following three pathways are the main mechanisms by which cKP, hvKP, and CRKP evolve into CR-hvKP.

#### Transfer of the plasmids carrying the carbapenem resistance genes into hvKP to form CR-hvKP

Plasmids, transposons, phages and insertion elements carrying movable carbapenem resistance genes in CRKP can be horizontally transferred to hvKP strains to form CR-hvKP. Because of the regional distribution characteristics of carbapenemases (Cui et al., 2019), the carbapenem resistance phenotype acquired by hvKP also showed similar regional characteristics. For example, KPC genes were the most common carbapenemase genes in China and the United States (Cui et al., 2019), and KPC-positive CR-hvKP strains were also reported more frequently in these areas. In 2014, a study in the United States was the first to report a hypermucoviscous ST23 KP strain that acquired the *bla_KPC-2_* gene and evolved into multidrug-resistant CR-hvKP (Cejas et al., 2014). Similarly, five K1 hvKP strains were reported in China, which formed K1 CR-hvKP by acquiring virulence plasmids harboring the *bla_KPC-2_* gene or combining a movable DNA fragment carrying *bla_KPC-2_* (Zhang et al., 2016). Furthermore, similar evolutionary mechanisms were also mentioned in a study in Hong Kong, China (Dong et al., 2019). HvKP strains with ST23 and K1 capsule serotypes acquired *bla_VIM-1_*-bearing carbapenem resistance plasmids via horizontal transfer, thus evolving into CR-hvKP (Dong et al., 2019). In addition, NDM and OXA are more common in Russia and India. In 2018, Russian researchers first reported that ST23 and K1 hvKP simultaneously acquired plasmids carrying the extended-spectrum β-lactamase CTX-M-15 gene and carbapenemase OXA-48 gene and evolved into CR-hvKP (Lev et al., 2018). In India, it was also reported that a hvKP strain harboring *bla_OXA_* and *bla_NDM_* genes encoding carbapenem resistance caused two deaths (Shankar et al., 2016). Notably, a study referenced a rare IMP-positive CR-hvKP isolate, XH210, recovered from human blood in Hangzhou, China (He et al., 2022). This CR-hvKP strain was characterized as having the ST17 KL38/O2 serotype and had the resistance plasmid pXH210-IMP, which carries the *bla_IMP-4_* gene. pXH210-IMP could be transferred from XH210 to *E. coli* and KP recipients in a conjugation experiment, indicating that the resistance plasmid had transferability. Furthermore, XH210 exhibited hypervirulence in the *Galleria mellonella* and mouse infection models but lacked the characteristic markers that are frequently associated with hypervirulence. The study demonstrated that hvKP strains without an obvious hypervirulence phenotype could evolve into CR-hvPK by acquiring the *bla_IMP-4_* gene (He et al., 2022). The horizontal transmission of mobile genes is the key factor leading to the continuous evolution of KP into CR-hvKP (Chen et al., 2014). The acquisition of carbapenem resistance genes by these virulent strains indicates that these major hospital pathogens are evolving.

#### CRKP acquired virulence plasmids and evolved into CR-hvKP

Compared with highly virulent strains, multidrug-resistant strains are more likely to produce a wide range of surface polysaccharide sites by chromosomal recombination and are more likely to acquire virulence plasmids (Wyres et al., 2019). Therefore, the original CRKP easily evolved into CR-hvKP through horizontal transfer of mobile virulence genes. A number of studies have shown that the virulence of CRKP can be significantly enhanced after acquisition of the pLVPK/pLVPK-like virulence plasmid (Gu et al., 2018a; Zhou et al., 2020; Zhang et al., 2020b). For example, an outbreak of ST11 CR-hvKP was reported in a hospital in China in 2018 (Gu et al., 2018a). Five patients died due to severe pneumonia after CR-hvKP infection. The researchers found that all the CR-hvKP isolates were positive in the string test and showed high virulence in a neutrophil killing assay and *Galleria mellonella* infection model (Gu et al., 2018a). Furthermore, these CR-hvKP isolates all carried a 170 kb pLVPK-like virulence plasmid (Gu et al., 2018a). The virulence plasmid of hvKP can be horizontally transferred to ST11 CRKP alone or can lead to the formation of ST11 CR-hvKP by conjugating with the IncF plasmid (Xu et al., 2021b). Interestingly, Bolourchi et al. (2021) claims that two CR-hvKP strains evolved from acquiring the plasmids carrying carbapenem resistance genes by hvKP strains carrying non-conjugated virulence plasmids. According to the latest study, non-conjugated virulence plasmids can integrate other plasmids carrying conjugative transfer genes to improve their own transmission. When this new conjugation plasmid was transferred to *E. coli* strain EC600 or other types of KP, its virulence potential in the mouse infection model could be significantly improved (Yang et al., 2022a). After acquiring the virulence plasmid, these CR-hvKP strains presented strong virulence and had the capacity of high virulence transmission (Xu et al., 2021b), which is more likely to cause outbreaks in hospitals or communities.

#### KP acquired the fusion plasmid with both virulence and carbapenem resistance genes to form CR-hvKP

In recent years, many studies have reported the evolution of KP into CR-hvKP via the acquisition of hybrid plasmids. There are two important mechanisms for the formation of hybrid plasmids. One involves two single-chain fragments changing at special fusion sites to form hybrid plasmids, and the other involves homologous recombination to form hybrid plasmids (Xu et al., 2021b; Figure 2).

Researchers have detected the fusion plasmid p17ZR-91-Vir-KPC in an ST86, K2 CR-HvKP strain in China (Xie et al., 2021). The p17ZR-91-Vir-KPC plasmid was formed by homologous recombination of the pLVPK-like virulence plasmid p17ZR-91-Vir and the circular resistance plasmid p17ZR-91-KPC carrying *bla_KPC-2_* in HR1 and HR2 homologous regions. Notably, the p17ZR-91-Vir-KPC fusion plasmid can be transferred among strains with different sequence types. ST11 hvKP can evolve into ST11 CR-hvKP by acquiring the fusion plasmid, which is an evolutionary mechanism for the most prevalent strain ST11 CR-hvKP (Xie et al., 2021). In addition, one study found a hybrid plasmid, pCRHV-C2244, carrying a series of virulence genes, including *iroBCDN*, *iucABCD*-*iutA,* and *rmpA2*, and the carbapenem resistance gene *bla_KPC-2_,* which was detected in an ST11, K64 CRKP strain in China in 2021. Multiple IS*26* sequences on the plasmid can regulate the recombination of the fragments carrying *bla_KPC-2_* and virulence genes, reverse the large sequence fragment on the plasmid, and finally form the hybrid plasmid pCRHV-C2244 (Jin et al., 2021). Thus, recombination between gene fragments with insertion elements is a mechanism for the formation of hybrid plasmids.

In addition, the virulence plasmid can be integrated into chromosomes, allowing bacteria to evolve into a more virulent strain. In 2021, two hvKP strains were isolated from the wound of a severely infected patient (Eger et al., 2021). Whole-genome sequencing revealed that the hypervirulence genes, such as *iroBCDN*, *iucABCD/iutA*, *rmpA/A2* and *peg,* were all located in the specific regions inserted into the chromosome. This suggests that the virulence plasmids can be integrated into the chromosome. Further analysis showed that these processes affected the function of chromosomes, and the evolved highly virulent strains could be vertically transmitted (Eger et al., 2021). The virulence plasmids integrated into chromosomes of the above KP strains indicated that hvKP can evolve by transferring its genes horizontally to other strains and can evolve through vertical transmission.

#### High-risk clone: ST11

The recent emergence of ST11 is the most frequent clone for CR-hvKP globally, especially in China (Wu et al., 2017; Liao et al., 2020; Wei et al., 2022). ST11 CR-hvKP can cause bacterial liver abscess (Yang et al., 2020; Cai et al., 2022), bacteremia and other infections, and it is a high-risk clinical pathogen that attracts worldwide attention (Wu et al., 2017). Notably, a report from China showed that pneumonia with high mortality was caused by ST11 CR-hvKP (Yang et al., 2022b). Hybrid conjugative virulence plasmids, e.g., a pLVPK-like hybrid plasmid with high virulence and carbapenem resistance genes, are demonstrated to readily convert an ST11 CRKP strain to a CR-hvKP strain via conjugation (Xie et al., 2021; Yang et al., 2022b). Therefore, ST11 CR-hvKP is a challenge for clinicians in various clinical settings and deserves more attention (Gu et al., 2018a).

## The epidemiology of CR-hvKP

In recent years, CR-hvKP has been increasingly reported in China (Gu et al., 2018a; Yuan et al., 2019; Su et al., 2020; Yang et al., 2020; Zhang et al., 2020b; Chen and Chen, 2021), the United States (Chiang et al., 2016), India (Shankar et al., 2016), Russia (Shaidullina et al., 2020), Egypt (Ahmed et al., 2021), Italy (Di Pilato et al., 2021), and other countries, represented by the ST11, ST23, and ST258 types. In this study, we summarized the relevant information on the CR-hvKP strains identified in articles published from June 2015 to April 2022. As shown in Table 1, Asia is the main epidemic area of CR-hvKP, represented by China, India and Singapore. China reported the largest number of CR-hvKP strains among these countries, and the most common sequence type was ST11 (Zhang et al., 2017), followed by ST23 (Su et al., 2021), ST25 (Li et al., 2019b) and ST65 (Su et al., 2021). The most common capsular serotypes were K1 (Zhang et al., 2016) and K2 (Su et al., 2020), followed by K64 (Hao et al., 2020). In China, the *bla_KPC-2_* gene in CR-hvKP was widespread, and the *bla_KPC_* gene detection rate was as high as 87.3% (715/819), while the detection rates of the *bla_NDM_*, *bla_OXA_*, *bla_IMP_* and *bla_VIM_* genes were 3.3% (27/819), 1.3% (11/819), 0.3% (3/819), and 0.2% (2/819), respectively (Table 1). CR-hvKP strains that carry two or more carbapenem resistance genes were also found in China (7.6%, 61/819). In other Asian countries, such as India (Shankar et al., 2016) and Iran (Mohammad Ali Tabrizi et al., 2018; Solgi et al., 2020), *bla_OXA_* and *bla_NDM_* genes were more common. In Iran, the detection rates of *bla_OXA_* and *bla_NDM_* were 54.9% (73/133) and 7.6% (10/133), respectively. In Japan (Yonekawa et al., 2020), the main detected enzyme was IMP, and the detection rate was 100% (42/42). The virulence plasmids in CR-hvKP strains were usually reported in China, and the most widespread were the pLVPK (Gu et al., 2018a) and pLVPK-like plasmids (Li et al., 2020b; Chen et al., 2021). In addition, hybrid plasmids pKP70-2 (Dong et al., 2018) and pCRHV-C2244 (Jin et al., 2021) were also reported. Europe and America have the second highest prevalence of CR-hvKP, mainly represented by ST23 and K1/K2 capsular serotypes strains. The primary carbapenem resistance genes in Europe are *bla_NDM_* and *bla_OXA_* (Becker et al., 2018; Lev et al., 2018; Turton et al., 2018), while *bla_KPC-2_* is more prevalent in America (Karlsson et al., 2019; Mataseje et al., 2019). The pLVPK-like virulence plasmids have also been reported in Europe and the United States (Lev et al., 2018), but there are few reports on the fusion plasmid with both virulence and carbapenem resistance genes. The virulence genes of these CR-hvKP strains are usually located on chromosomes, with few strains carrying virulence plasmids. Moreover, two cases of ST11-type CR-hvKP infection were reported in Egypt in the last 2 years. One strain carried both *bla_KPC-2_* and *bla_NDM-1_* genes (Ahmed et al., 2021), while the other strain carried both *bla_KPC-2_* and *bla_OXA-48_* genes (Yang et al., 2022c). In 2020, 10 CR-hvKP strains carrying both *bla_NDM_* and *bla_OXA-48_* genes were reported in Sudan (Albasha et al., 2020).

## The treatment of CR-hvKP

As a class of bacteria of CRE, most of the current treatment plans for CR-hvKP refer to the treatment guidelines of CRE. Currently, carbapenems are the most widely used first-line antibiotics for CRE (Tzouvelekis et al., 2012). Meropenem, meropenem-vaborbactam and imipenem-relebactam are commonly used in pediatric CRE infection diseases (Chiotos et al., 2020). Overwise, meropenem-vaborbactam can effectively inhibit the growth of CRE strain producing KPC, and has a higher clinical cure rate and lower side effects, especially nephrotoxicity (Pfaller et al., 2018). Pascale et al. noted that high-dose continuous infusion of meropenem is a wise choice to treat CRE in exceptional cases (Pascale et al., 2019). However, the use of carbapenem antibiotics leads to an increasing number of carbapenemase-producing strains and increases the difficulty of clinical treatment (Tzouvelekis et al., 2012). Some new anti-CRE antibacterials are also good options for CRE treatment, including ceftazidime-avibactam (Shields et al., 2018; Chiotos et al., 2020), plazomicin, tigecycline combination therapy (Ni et al., 2016) and colistin (Li et al., 2019a). The use of ceftazidime-avibactam against CRKP strains has shown an excellent therapeutic effect on clinical cure rate and survival rate (Farrell et al., 2014; Yin et al., 2019). However, high ceftazidime hydrolysis activity and OmpK35 porin deficiency can lead to the decreased susceptibility to ceftazidime-avibactam in KPC-producing ST11 CRKP (Shen et al., 2017). Importantly, given the different epidemiological situations in different regions and the characteristics of enzyme production of the strains, clinical medication should be based on specific circumstances (Pogue et al., 2019).

## Conclusion

CR-hvKP has become widespread in many countries. Treatment options for CR-hvKP are also relatively limited. The transfer of plasmids carrying removable genes, as well as the fusion and recombination of plasmids between bacteria, are the key factors underlying the high mutation and transmission rates of CR-hvKP. Future studies should also pay more attention to the vertical transmission of the virulence plasmid of CR-hvKP integrated into the chromosome, which may lead to new discoveries in research on the molecular mechanisms of CR-hvKP.

## Author contributions

W-QZ designed and supervised the study. Y-LH collected the data from previous studies and drafted the manuscript. X-HW, X-SC, WZ, and J-XW provided administrative support and provided intellectual contributions to the manuscript. J-RW, Z-DH, and W-QZ critically reviewed and edited the manuscript. All authors contributed to the article and approved the submitted version.

## Funding

This work was supported by the General Program of the Inner Mongolia Medical University Inner Mongolia (YKD2021MS011), the Young Talents of Science and Technology in Universities of Inner Mongolia (NJYT-20-B14), and the Science and Technology innovation team of the Inner Mongolia Medical University Inner Mongolia.

## Conflict of interest

The authors declare that the research was conducted in the absence of any commercial or financial relationships that could be construed as a potential conflict of interest.

## Publisher’s note

All claims expressed in this article are solely those of the authors and do not necessarily represent those of their affiliated organizations, or those of the publisher, the editors and the reviewers. Any product that may be evaluated in this article, or claim that may be made by its manufacturer, is not guaranteed or endorsed by the publisher.
