# Supplementary material for: Epidemiological characteristics and molecular evolution mechanisms of carbapenem-resistant hypervirulent Klebsiella pneumoniae
Source: Front Microbiol. 2022 Sep 12;13:1003783. doi: 10.3389/fmicb.2022.1003783 (PMC9524375; doi:10.3389/fmicb.2022.1003783)
Supplement: Supplementary file 1 [file Table_1.DOCX]

**Supplementary Table 1.** Summary of global reports on CR-hvKP from June 2015 to April 2022

|  | Country | Number of strains ^a^ | carbapenem resistance genes ^b^ | Virulence genes ^c^ | Virulence plasmid | Serotype | Sequence type | Year of report/year of the strain collection | References |
| --- | --- | --- | --- | --- | --- | --- | --- | --- | --- |
| Asia | China | 6 | *bla_KPC-2_* | *repA, fimH, uge, wabG, ureA, entB, ybtS, iroN, mrkD, ycfM* | - | K2/ non-typeable | ST11, ST25, ST65 | 2015/ 2010-2014 | (Yao et al., 2015) |
|  | China | 3 | *bla_IMP-4_*, *bla_KPC-2_* | *rmpA, entB, ybtS, fimH, mrkD*, *aerobactin* | - | K2/ non-typeable | ST11, ST1700 | 2015/ 2012-2013 | (Zhang et al., 2015) |
|  | China | 1 | *bla_NDM-5_* | *kfuABC*, *mrkABCDFHIJ*, *rmpA* | - | K2 | ST14 | 2016/ 2015 | (Liu et al., 2016) |
|  | China | 5 | *bla_KPC-2_* | *magA,, rmpA, wcaG* | A 200-kb plasmid | K1 | ST23, ST1797 | 2016/ 2013 | (Zhang et al., 2016) |
|  | China | 7 | *bla_KPC-2_* | *fimH-1*, *mrkD*, *magA*, *rmpA*, *rmpA2*, *iroN*, *allS*, *aerobactin*, *traT*, *iutA* | - | K1, K2, K8, K37, K47, K64 | ST11, ST15, ST23, ST65, ST147, ST1456 | 2017 /2012-2015 | (Liu et al., 2017) |
|  | China | 21 | *bla_KPC-2_* | *ureA, wabG, fimH, entB, ycf, ybtS, iutA, rmpA, iroNB, kfu, wcaG* | - | K2, K20/ non-typeable | ST11, ST65, ST268, ST595, ST692 | 2017 /2013-2015 | (Zhan et al., 2017) |
|  | China | 1 | *bla_KPC-2_* | *rmpA2* | pKP70-2（Integrated plasmid carrying *bla_KPC-2_* and *rmpA2*） | K1 | ST23 | 2018/ 2013 | (Dong et al., 2018) |
|  | China | 1 | *bla_KPC-2_* | *rmpA, rmpA2*, *iucABCD-iutA, clbA-R, iroBCDN, fyuA, irp2, ybtAEPQTUX, mrkABCDFHIJ* | pVir-KP13F2 | K62 | ST36 | 2018/2015 | (Feng et al., 2018) |
|  | China | 5 | *bla_KPC-2_* | *entB, fimABCDEFGHIK, iroE, irp1/2, iucABCD-iutA, kpn, mrkABCDFHIJ, rmpA2, ycfM* | pVir-CR-HvKP4 | - | ST11 | 2018/ 2016 | (Gu et al., 2018a) |
|  | China | 2 | *bla_KPC-2_* | *rmpA, iucABCD, iroBCDN* | p227035-3 | - | ST11 | 2018/ 2017 | (Gu et al., 2018b) |
|  | China | 1 | *bla_KPC-2_* | *iroBCDN**, iucABCD-iutA, rmpA, rmpA2* | TVGHCRE225 pVir | K47 | ST11 | 2018/ 2012-2014 | (Huang et al., 2018) |
|  | China | 1 | *bla_KPC-2_*, *bla_NDM-1_* | *rmpA, rmpA2, iroN, aerobactin, mrkD* | - | K2 | ST86 | 2018/ 2016 | (Wei et al., 2018) |
|  | China | 1 | *bla_VIM-1_* | *mrkABCDFJIH*, *clbABCDEFGHIJKLMNOPQR*, *fyuA*, *irp1/2*, *ybtSXQPA*, *mceABCDEJIHG*, *kfuABC*, *arcC*, *allABCDRS*, *glxKR*, *iutA-iucABCD*, *iroBCDN*, *rmpA*, *rmpA2* | pR210-2-vir | K1 | ST23 | 2019/ 2018 | (Dong et al., 2019) |
|  | China | 14 | *bla_KPC-2,_ bla_NDM-1_* | *rmpA*, *iucB*, *iroB*, *uge*, *wabG*, *ureA*, *allS*, *ybtS*, *fimH*, *entB* | - | K2/ non-typeable | ST25, ST11 | 2019/ 2014-2017 | (Li et al., 2019) |
|  | China | 1 | *bla_NDM-1_*, *bla_OXA-1_* | *rmpA*, *rmpA2*, *iucABCD-iutA*, *iroBCDN* | pVIR_3214 | K1 | ST23 | 2019/ - | (Liu and Su, 2019) |
|  | China | 13 | *bla_KPC-2_*, *bla_NDM-1_* | *allS*, *rmpA*, *aerobactin*, *iroB*, *entB*, *iucA*, *kfuBC*, *mrkD*, *rmpA2*, *ureA*, *wabG*, *ybtS*, *ycfM* | - | K47, K64 | ST11, ST1764 | 2019/2013-2017 | (Liu et al., 2019) |
|  | China | 1 | *bla_NDM-5_* | *iucABCD-iutA*, *iroBCDN*, *rmpA*, *fimACDGH*, *entABCEF* | - | K108 | ST35 | 2019/ 2016-2018 | (Shen et al., 2019) |
|  | China | 9 | *bla_OXA-232_* | *iucABCD-iutA*, *rmpA2*, *ybtAEPQSTUX*, *fyuA*, *irp1*/*2*, *mrkABCDFHIJ*, *kfuABC* | pE109-1-vir | KL112 | ST15 | 2019/ 2018 | (Shu et al., 2019) |
|  | China | 15 | *bla_KPC-2_* | *magA, allS*, *kfu*, *fimH*, *wabG*, *ybts*, *mrkD*, *uge*, *entB*, *iutA*, *rmpA*, *rmpA2*, *iucA*, *iroN* | - | K1, K16, K47, K64, | ST11, ST23, ST660, ST1660 | 2019/ 2011-2017 | (Xu et al., 2019) |
|  | China | 1 | *bla_NDM-5_* | *iucABCD-iutA*, *iroBCDEN*, *rmpA*, *entABCDEFS*, *fepABCDG*, *fyuA*, *irp1/2*, *ybtAEPQSTUX*, *mrkABCDFHIJ* | pVir-SCNJ1 | K54 | ST29 | 2019/ 2018 | (Yuan et al., 2019) |
|  | China | 1 | *bla_KPC-2_* | *iucABCD*, *rmpA2*, *iroBCD* | - | - | ST11 | 2019/ 2016 | (Zhou et al., 2019) |
|  | China | 31 | *bla_KPC-2_*, *bla_OXA-1_* | *peg-344*, *iucA*, *rmpA*, *rmpA2* | - | KL64, KL47 | ST11 | 2020/ 2017-2018 | (Liu et al., 2020) |
|  | China | 1 | *bla_KPC-2_* | *mrkA-K*, *clbA-Q*, *fyuA*, *ybtAEPQSTUX*, *irp1/2*, *kfuABC, hisl, cysS, cobW, dskA, folE, queF, sitABCD, thrS, pyrC, hemB, znuAC, mceBCDJHG, arc, ylbFE, fdrA, allDC, glxK, ybbWY, allABRS, KP1, glxR, hyi, gcl* | p11420-vir | K1 | ST1265 | 2020/ 2014 | (Li et al., 2020a) |
|  | China | 43 | *bla_KPC-2_*, *bla_NDM_* , *bla_IMP-4_* | *uge*, *mrkD*, *fimH*, *kpn*, *aerobactin*, *rmpA, allS* | tig00000014 | K2/ non-typeable | ST11, ST76, ST375, ST323, ST530, ST896, ST2964 ST3335 | 2020/ 2015-2017 | (Su et al., 2020) |
|  | China | 8 | *bla_KPC-2_*, *bla_KPC-5_* | *iucABCD-iutA,* *ybtAEPQSTUX,* *iroBCDN*, *mrkABCDFHIJ*, *kfu*, *irp1/2*, *fyuA* | - | K47, K64 | ST11 | 2020/ 2012-2016 | (Yang et al., 2020) |
|  | China | 55 | *bla_KPC-2_*, *bla_NDM-1_* | *iucA*, *iroN*, *rmpA*, *rmpA2* | pVir-CRhvKP-C789, pVir-CR-hvKP-C1398 | K2, K57, K64 | ST11 | 2020/ 2015-2017 | (Zhang et al., 2020) |
|  | China | 152 | *bla_KPC_*_-2_, *bla_OXA-1_* | *ybtAEPQSTUX*, *irp1/2*, *fyuA*, *rmpA*, *rmpA2*, *iucABCD-iutA*, *peg-344*, *iroBCDN* | pVir-KP16932, pVir-KP47434 | KL47, KL64, KL103, KL105, KL31 | ST11 | 2020/ 2013-2017 | (Zhou et al., 2020) |
|  | China | 1 | *bla_KPC-2_* | *rmpA2*, enterobactin, yersiniabactin, salmochelin | - | K64 | ST11 | 2020/ 2017 | (Jin et al., 2020) |
|  | China | 19 | *bla_KPC-2_* | *rmpA*, *rmpA2*, *irp1/2*, *iucABCD-iutA*, *mrkABCDFHIJ*, *ybtAEPQTUX*, *fyuA* | IncHI1B plasmid | KL47, K64 | ST11 | 2020/ 2016-2017 | (Zheng et al., 2020) |
|  | China | 9 | *bla_KPC-2_*_,_ *bla_NDM-1_* , *bla_NDM-5_* | rmpA2, *terW*, *iutA*, *silS* | pLVPK-like plasmid | K1, K2, K64 | ST11, ST23, ST86 | 2020/ 2016-2018 | (Li et al., 2020b) |
|  | China | 84 | *bla_KPC-2_* | *iutA,* *rmpA*, *rmpA2* | - | KL47, K64 | ST11, ST65, ST268, ST412, ST595 | 2020/ 2014-2019 | (Hao et al., 2020) |
|  | China | 1 | *bla_KPC-_*_2_ | *entABCDEFS*, *fepABCDG*, *fimABCDEFHIK*, *iroEN*, *iutA*, *mrkABCDFHIJ*, *iroBCDN*, *pagO*, *rmpA* | pK55602_1 | K2 | ST86 | 2021/ 2017 | (Liu et al., 2021) |
|  | China | 1 | *bla_KPC-_*_2_ | *iucABCD-iutA*, *iroBCDEN*, *rmpA*, *rmpA2*, *ybt9* , *mrkABCDFHIJ* | p17ZR-91-Vir | K2 | ST86 | 2021/ 2017 | (Xie et al., 2021) |
|  | China | 1 | *bla_KPC-2_* | *allABCRS*, *clbABCDEFGHIJKLMNOPQR*, *fyuA*, *irp1*/*2*, *ybtAEPQSTUX*, *mrkABCDFHIJ*, *kfuABC*, *arc*, *fdrA*, *gcl*, *glxKR*, *ylbEF* | - | K1 | ST23 | 2021/ 2017 | (Yan et al., 2021) |
|  | China | 1 | *bla_KPC-2_* | *rmpA2*, *iroBCDN*, *iucABCD-iutA* | pCRHV-C2244(Integrated plasmid carrying *bla_KPC-2_* and *rmpA2*, *iroBCDN*, *iucABCD-iutA*) | K64 | ST11 | 2021/ 2017 | (Jin et al., 2021) |
|  | China | 8 | *bla_KPC_* | *rmpA, rmpA2, iucA, iutA* | - | K14/K64/ non-typeable | ST11 | 2021/ 2017-2019 | (Li et al., 2021a) |
|  | China | 32 | *bla_KPC-2_*, *bla_NDM-1_*, *bla_OXA-1_* | yersiniabactin, aerobactin, salmonchelin, *rmpA*, *rmpA2* | pLVPK-like plasmid | KL64, KL10, KL25, KL47, KL62, KL2, KL19, KL24 | ST11, ST37, ST15, ST304, ST2237 | 2021/ 2018-2019 | (Chen et al., 2021) |
|  | China | 1 | *bla_KPC-2_* | *rmpA2*, *iucABCD-iutA* | pSH12_Vir | KL47 | ST11 | 2021/ 2015 | (Yang et al., 2021) |
|  | China | 1 | *bla_KPC-2_* | *rmpA*, *rmpA2*, *iucABCD* | pVir-C2582 | K64 | ST11 | 2021/ 2017 | (Zhang et al., 2021) |
|  | China | 39 | *bla_KPC-2_*, *bla_NDM-1_* | *rmpA*, *terW*, *silS*, *iutA*, *rmpA2* *kpn*, *entB*, *ytbs*, *repA*, *aerobactin*, *magA*, *kfuBC*, *wcaG* | pLVPK-like plasmid | K1, K2/ non-typeable | ST11, ST23, ST65, ST86 | 2021/ 2017-2018 | (Liao et al., 2021) |
|  | China | 45 | *bla_KPC-2_*, *bla_NDM-1_*, *bla_VIM-2_* | *rmpA*, *terW*, *silS*, *iutA*, *rmpA2* | pLVPK-like plasmid | - | ST11, ST23 | 2021/ 2018-2019 | (Huang et al., 2021) |
|  | China | 32 | *bla_KPC-2_* | *fimH, iucB, mrkD, rmpA, uge, wabG* | - | K57 | ST11 | 2021/ 2019 | (Shao et al., 2021) |
|  | China | 1 | *bla_NDM-5_* | *rmpA*, *iucABCD-iutA*, *iroBCDN* | pVir22937 | K2 | ST65 | 2021/ 2019 | (Zhao et al., 2021) |
|  | China | 12 | *bla_KPC-2_*, *bla_NDM-1_*, *bla_VIM-1_*, *bla_OXA-48_* | *rmpA*, *rmpA2* | - | K2, K14/K64 | ST25, ST11 | 2021/ 2019-2020 | (Su et al., 2021) |
|  | China | 3 | *bla_NDM-1_, bla_OXA-1_, bla_VIM-1_* | *_p_rmpA*, *_p_rmpA2*, *_c_rmpA*, *peg-344*, *iroB*, *ytbS*, *mrkD*,  *entB*, *kfu*,  *allS*, *iutA*,  *K_2_A,*  *wabG*,  *uge*, *fimH*,  *wcaG*, *kpn*, *ycfM*,  *iroN* | - | K2, K57, K54 | ST29, ST14, ST17, ST37, ST45, ST101, ST234 | 2021/ 2016-2020 | (Li et al., 2021b) |
|  | China | 51 | *bla_KPC-2_* | *rmpA*, *rmpA2*, *peg344*, *iucA*, *iroN* | - | K64, K54/ non-typeable | ST11, ST15, ST198 | 2022/ 2020 | (Wei et al., 2022) |
|  | China | 60 | *bla_KPC-2_* | *rmpA*, *rmpA2*, *iutA* | pLVPK-like plasmid | KL64, KL20, KL47, KL16, KL19, KL62, KL3, KL107, KL28, KL36, KL2, KL21 | ST11, ST268, ST595, ST395, ST437, ST86, ST2245-1LV, ST290 | 2022/ 2014-2017 | (Yang et al., 2022a) |
|  | China | 16 | *bla_KPC-2_* | *aerobactin* , *entB*, *allS*, *mrkD*, *fimH*, *rmpA* , *rmpA2*,  *iutA*, *iucA* | - | K1, K2, K47, K64 | ST11, ST23, ST86 | 2022/ 2019-2020 | (Zhou et al., 2021) |
|  | China | 1 | *bla_KPC_* | *rmpA*, *iutA* | - | - | - | 2022/ 2021 | (Zhou et al., 2022) |
|  | India | 3 | *bla_OXA-1_*, *bla_OXA-181_*, *bla_OXA-232_*, *bla_NDM-1_* | *mrkD, iutA-iucABCD, entADEF, ybtAUT, irp1/2, kfuABC, MviM*, *fyuA*, *rmpA2* | - | - | ST11, ST43, ST231 | 2016/ 2016 | (Shankar et al., 2016) |
|  | India | 1 | *bla_OXA-232_* | *mrkABCDFHIJ*, *kfuABC*, *iucABCD-iutA*, *iroBCDEN*, *rmpA*, *rmpA2*, *rcsAB*, *entABCDEFS*, *fepABCDG*, *fes*, *fyuA*, *irp1*, *irp2*, *ybtAEPQSTUX*, *allABCDRS* | - | K1 | ST23 | 2020/ 2016 | (Mukherjee et al., 2020) |
|  | Iran | 5 | *bla_VIM-2_* | *rmpA, magA, iucABCD-iutA* | - | K1 | ST23 | 2018/ 2012 | (Mohammad Ali Tabrizi et al., 2018) |
|  | Iran | 3 | - | *peg344*, *iucA*, *iroB*, *rmpA*, *rmpA2*, *iutA*, *mrkD*, *ybtS* | - | K1, K2 | ST147, ST392 | 2020/ 2015-2016 | (Pajand et al., 2020) |
|  | Iran | 52 | *bla_NDM -1_*, *bla_NDM-7_*, *bla_OXA-48_* | *iucA*, *iroN*, *rmpA*, *ybtS*, *ycf*, *magA*, *mrkD* | - | K1, K2/ non-typeable | ST11, ST893, ST147, ST15, ST392 | 2020/ 2017-2018 | (Solgi et al., 2020) |
|  | Iran | 4 | *bla_NDM_*, *bla_OXA-48_* | *rmpA/A2，fyuA， kfuB，ybtAEQSU，iroC，* *irp1/2，iucA* | - | - | ST5235 | 2020/2017 | (Banerjee et al., 2021) |
|  | Iran | 59 | *bla_NDM-1_*, *bla_OXA-48_* | *iucA*, *iutA*, *ybt*, *rmpA*, *kfu*, *peg344*, *iro*, *magA*, *allS* | - | K2, K20 | - | 2021/ 2019-2020 | (Sanikhani et al., 2021) |
|  | Iran | 13 | *bla_OXA-1_*, *bla_OXA-48,_* | *iutABCD*, *iucA, ybtAEPQSTUX, fyu， irp1/2* | P35 plasmid 3, P38 plasmid 1 | K15， K17, K20, K107 | ST11, ST101, ST147, ST893 | 2021/2014-2016 | (Bolourchi et al., 2021) |
|  | Singapore | 32 | *bla_KPC-2_* | *ybt*, *clb*, *iuc*, *iro*, *rmpA*, *rmpA2* | - | KL1, KL2, KL25, K25, K47, K51, K107, K117, K124, K136 | ST11, ST14, ST23, ST17, ST48, ST65, ST70, ST86, ST231, ST273, ST792, ST1117 | 2019/ 2011-2015 | (Octavia et al., 2019) |
|  | Singapore | 18 | *bla_KPC-2_*，*bla_OXA-1_* | *rmpA*, *rmpA2*, *ybt, clb*, *iuc*, *iro* | - | K1, K2, K20 | ST23, ST65, ST86, ST420, ST893 | 2020/ 2010-2015 | (Chen et al., 2020) |
|  | Japan | 1 | *bla_IMP-6_* | *iucABCD-iutA*, *iroBCDEN*, *rmpA*, *rmpA2* | pTHC11-1 | K1 | ST23 | 2019/ 2012 | (Harada et al., 2019) |
|  | Japan | 41 | *bla_IMP_* | *rmpA/A2, iroN, iutA* | - | K1, k2 | ST23, ST65, ST86, other ST | 2020/2014-2015 | (Yonekawa et al., 2020) |
| Europe | Russia | 20 | *bla_OXA-48_*, *bla_OXA-244,_  bla_NDM-1_* | *rmpA, aerobactin, uge2, wabG, kfu, fimH, allR* | pLVPK-like plasmid | K1, K2, K57, *wzi420^c^* | ST23, ST147, ST218, ST395, ST833, ST20 | 2018/ 2012-2016 | (Lev et al., 2018) |
|  | Russia | 1 | *bla_OXA-48_*, *bla_OXA-1_* | *mrkABCDFHIJ*, *allABCDRS*, *mceABCDEGHIJ*, *ybtAEPQSUX*, *fyuA*, *irp1*, *kfuABC*, *iucABCD*, *iroBCDN*, *rmpA*, *rmpA2* | IncHI1B/ FIB plasmid | K1 | ST23 | 2020/ 2014 | (Shaidullina et al., 2020) |
|  | Russia | 9 | *bla_NDM-1_*, *bla_NDM-29_*, *bla_OXA-_**_1_*, *bla_OXA-9_* | *entABES*, *fepBCDG*, *ecpABCDER*, *fimABCEH*, *ybtAEPQSTUX*, *fyuA*, *irp1/2*, *kdsA*, *_p_rmpA, _p_rmpA2*, *iucABCD*, *iutA*, *terABCDEWXYZ*, *peg-344*, *pagO*, *shiF*, *ydjA*, *cobW*, *luxR*, *arsABCDR*, *pcoABCDERS*, *silABCEF* | IncFI/ IncHI1B/ ColRNAI plasmid | KL2, KL19, KL20, KL45 | ST15, ST147, ST395, ST874 | 2021/ 2017-2019 | (Starkova et al., 2021) |
|  | Italy | 1 | *bla_NDM-5_*, *bla_OXA-48_*, *bla_OXA-9_* | *iucABCD-iutA*, *iroEN*, *rmpA*, *rmpA2*, *fimABCDEFGHIK*, *mrkABCDFIJ*, *pilW*, *entABCEDEFS*, *fepABCDG*, *fes*, *rcsAB*, *stbABCD* | pLVPK-like plasmid | KL30 | ST383 | 2020/ 2019 | (Scaltriti et al., 2020) |
|  | Italy | 4 | *bla_NDM-1_*, *bla_NDM-5_*, *bla_OXA-48_*, *bla_OXA-1_*, *bla_NDM-1_* | *iucABCD-iutA*, *rmpA*, *rmpA2*, *terABCDEWXYZ*, *mrkABCDF*, *irp*, *fyuA*, *kfuA* | IncFIB(pNDM-Mar)/ IncHIB (pNDM-MAR) _NDM5 (Integrated plasmid carrying *bla_NDM-5_* and *iucABCD-iutA*, *qnrS*, *rmpA*, *rmpA2*, *terABCDEWXYZ*) | KL51, KL64, KL112 | ST147, ST15, ST383 | 2022/ 2019 | (Lorenzin et al., 2022) |
|  | United Kingdom | 1 | *bla_NDM_* | *wcaG, allS, iucABCD-iutA, kfuABC, ybtAEUPQRSTX, clbABCDEFGHIJKLMNOPQR, iroBCDN, terABCDEWXZ, rmpA2* | - | K1 | ST23 | 2018/ 2015 | (Roulston et al., 2018) |
|  | United Kingdom | 16 | *bla_NDM-1_, bla_NDM-5_,* *bla_OXA-48_* | *rmpA, rmpA2,* *ybtS, mrkD, iutA*, *allS*, *terWXY, clbA-R,* , *iroBCDN, kfuABC*, *mrkABCDF*, *ybtAEPQSTUX*, *terABCDEWXY*, *silCERS*, *mceABCDEGHIJ*, *pbrABCR* | KpvST147, KpvST23L | K1/ non-typeable | ST23 | 2018/ 2016 | (Turton et al., 2018) |
|  | France | 1 | *bla_OXA-48_* | *rmpA*, *rmpA2*, *iucABCD-iutA*, *iroBCDN*, *ybt* | pVIR-Kpn154 | K2 | ST86 | 2020/ 2017 | (Beyrouthy et al., 2020) |
|  | Germany | 107 | *bla_OXA-48_, bla_KPC-3_,* *bla_KPC-2_*, *bla_NDM-1_* | *mrkABCDFHIJ*, yersiniabactin, aerobactin, *kfu* | - | K1 | ST11, ST15, ST16, ST17, ST23, ST37, ST48, ST101, ST147, ST258, ST307, ST340, ST347, ST391, ST395, ST512, ST629, ST784, ST906, ST1399, ST2254 | 2018/ 2008-2014 | (Becker et al., 2018) |
|  | Portugal | 1 | *bla_KPC-70_, bla_OXA-9_* | *fimA-K**, mrkA-J, iutA, iroN, iroE, fyuA–ybtX, clb-c* | - | KL3 | ST13 | 2022/ 2019 | (Mendes et al., 2022) |
| America | United States | 4 | *bla_OXA-9_, bla_KPC-3_,* *bla_KPC-2_* | *entABCDEF*, *iutA*, *iroN*, *kfuABC*, *fyuA*, *clbA-R*, *ybt*, *irp* complex, *mrkD*, *fimH* | - | K2, K51/ non-typeable | ST14, ST258, ST1082 | 2017/ 2012-2016 | (Krapp et al., 2017) |
|  | United States | 1 | *bla_KPC-2_*, *bla_OXA-9_* | *rmpA*, *rmpA2*, *iutA-iucABCD*, *iroBCDN*, *peg*-344, *allS*, *kfu, magA, mrkD, wcaG, ybtS* | pDHQP1701672_hv | K1 | ST23 | 2019/ 2016 | (Karlsson et al., 2019) |
|  | Canada | 2 | *bla_KPC-2_* | *rmpA*, *rmpA2*, *iroBCDN*, *iucABCD-iutA*, *ybtAEPQSTUX*, *fyuA*, *irp1*/*2* | phvKP060 | KL2 | ST86 | 2019/ 2018 | (Mataseje et al., 2019) |
|  | Colombia | 1 | *bla_KPC-2_* | *ybt*, *iuc*, *iro*, *rmpA* | - | KL2 | ST380 | 2021/ 2013 | (Saavedra et al., 2021) |
| Afica | Egypt | 1 | *bla_KPC-2_*, *bla_NDM-1_* | *fimABCDEFGHIK,* *mrkABCDFHIJ, rcsAB, galF, cpsACP, wzi, wza, gnd, ugd, ,sciN/tssJ, impA/tssA, tssGF, icmF/tssM, KPHS_23120, tle1, tli1, vgrG/tssI, clpV/tssD, dotU/tssL, vase/tssK, vipB/tssC, vipA/tssB, fepABCDG, fes, yagV/ecpE, yagW/ecpD, yagX/ecpC, yagY/ecpB, yagZ/ecpA, yagK/ecpR, fyuA, ybtAETUQXS, irp1/2, iroE, rmpA, rmpA2, iucABCD-iutA, entABCDEF,* | pEBSI036-1-NDM-VIR (Integrated plasmid carrying *bla_NDM-1_* and *iucABCD-iutA*, *rmpA*, *rmpA2*) | KL47 | ST11 | 2021/ 2017-2018 | (Ahmed et al., 2021) |
|  | Egypt | 1 | *bla_KPC-2_*, *bla_OXA-48_* | *iucABCD-iutA*, *rmpA*, *rmpA2* | pEBSI041-1 | - | ST11 | 2022/ 2012 | (Yang et al., 2022b) |
|  | Sudan | 10 | *bla_NDM_*, *bla_OXA-48_* | *mrkD*, *entB*, *rmpA*, *kfu*, *magA* | - | K2 | - | 2020/ 2017 | (Albasha et al., 2020) |

^a^ All strains came from the CR-hvKP-related articles reported from June 2015 to April 2022. The inclusion criteria for strains were: carried at least one carbapenem resistance gene, having two or more virulence genes or the string test was positive;

^b/c^ Carbapenem resistance genes and virulence genes carried by CR-hvKP strains were reported in the study.

**References**

Ahmed, M., Yang, Y., Yang, Y., Yan, B., Chen, G., Hassan, R.M., et al. (2021). Emergence of Hypervirulent Carbapenem-Resistant Klebsiella pneumoniae Coharboring a bla (NDM-1)-Carrying Virulent Plasmid and a bla (KPC-2)-Carrying Plasmid in an Egyptian Hospital. *mSphere* 6(3). doi: 10.1128/mSphere.00088-21.

Albasha, A.M., Osman, E.H., Abd-Alhalim, S., Alshaib, E.F., Al-Hassan, L., and Altayb, H.N. (2020). Detection of several carbapenems resistant and virulence genes in classical and hyper-virulent strains of Klebsiella pneumoniae isolated from hospitalized neonates and adults in Khartoum. *BMC Res Notes* 13(1)**,** 312. doi: 10.1186/s13104-020-05157-4.

Banerjee, T., Wangkheimayum, J., Sharma, S., Kumar, A., and Bhattacharjee, A. (2021). Extensively Drug-Resistant Hypervirulent Klebsiella pneumoniae From a Series of Neonatal Sepsis in a Tertiary Care Hospital, India. *Front Med (Lausanne)* 8**,** 645955. doi: 10.3389/fmed.2021.645955.

Becker, L., Kaase, M., Pfeifer, Y., Fuchs, S., Reuss, A., von Laer, A., et al. (2018). Genome-based analysis of Carbapenemase-producing Klebsiella pneumoniae isolates from German hospital patients, 2008-2014. *Antimicrob Resist Infect Control* 7**,** 62. doi: 10.1186/s13756-018-0352-y.

Beyrouthy, R., Dalmasso, G., Birer, A., Robin, F., and Bonnet, R. (2020). Carbapenem Resistance Conferred by OXA-48 in K2-ST86 Hypervirulent Klebsiella pneumoniae, France. *Emerg Infect Dis* 26(7)**,** 1529-1533. doi: 10.3201/eid2607.191490.

Bolourchi, N., Shahcheraghi, F., Giske, C.G., Nematzadeh, S., Noori Goodarzi, N., Solgi, H., et al. (2021). Comparative genome analysis of colistin-resistant OXA-48-producing Klebsiella pneumoniae clinical strains isolated from two Iranian hospitals. *Ann Clin Microbiol Antimicrob* 20(1)**,** 74. doi: 10.1186/s12941-021-00479-y.

Chen, R., Liu, Z., Xu, P., Qi, X., Qin, S., Wang, Z., et al. (2021). Deciphering the Epidemiological Characteristics and Molecular Features of bla KPC-2- or bla NDM-1-Positive Klebsiella pneumoniae Isolates in a Newly Established Hospital. *Front Microbiol* 12**,** 741093. doi: 10.3389/fmicb.2021.741093.

Chen, Y., Marimuthu, K., Teo, J., Venkatachalam, I., Cherng, B.P.Z., De Wang, L., et al. (2020). Acquisition of Plasmid with Carbapenem-Resistance Gene bla(KPC2) in Hypervirulent Klebsiella pneumoniae, Singapore. *Emerg Infect Dis* 26(3)**,** 549-559. doi: 10.3201/eid2603.191230.

Dong, N., Lin, D., Zhang, R., Chan, E.W., and Chen, S. (2018). Carriage of blaKPC-2 by a virulence plasmid in hypervirulent Klebsiella pneumoniae. *J Antimicrob Chemother* 73(12)**,** 3317-3321. doi: 10.1093/jac/dky358.

Dong, N., Sun, Q., Huang, Y., Shu, L., Ye, L., Zhang, R., et al. (2019). Evolution of Carbapenem-Resistant Serotype K1 Hypervirulent Klebsiella pneumoniae by Acquisition of bla (VIM-1)-Bearing Plasmid. *Antimicrob Agents Chemother* 63(9). doi: 10.1128/aac.01056-19.

Feng, Y., Lu, Y., Yao, Z., and Zong, Z. (2018). Carbapenem-Resistant Hypervirulent Klebsiella pneumoniae of Sequence Type 36. *Antimicrob Agents Chemother* 62(7). doi: 10.1128/aac.02644-17.

Gu, D., Dong, N., Zheng, Z., Lin, D., Huang, M., Wang, L., et al. (2018a). A fatal outbreak of ST11 carbapenem-resistant hypervirulent Klebsiella pneumoniae in a Chinese hospital: a molecular epidemiological study. *Lancet Infect Dis* 18(1)**,** 37-46. doi: 10.1016/S1473-3099(17)30489-9.

Gu, D., Lv, H., Sun, Q., Shu, L., and Zhang, R. (2018b). Emergence of tet(A) and bla(KPC-2) co-carrying plasmid from a ST11 hypervirulent Klebsiella pneumoniae isolate in patient's gut. *Int J Antimicrob Agents* 52(2)**,** 307-308. doi: 10.1016/j.ijantimicag.2018.06.003.

Hao, M., Shi, X., Lv, J., Niu, S., Cheng, S., Du, H., et al. (2020). In vitro Activity of Apramycin Against Carbapenem-Resistant and Hypervirulent Klebsiella pneumoniae Isolates. *Front Microbiol* 11**,** 425. doi: 10.3389/fmicb.2020.00425.

Harada, S., Aoki, K., Ishii, Y., Ohno, Y., Nakamura, A., Komatsu, M., et al. (2019). Emergence of IMP-producing hypervirulent Klebsiella pneumoniae carrying a pLVPK-like virulence plasmid. *Int J Antimicrob Agents* 53(6)**,** 873-875. doi: 10.1016/j.ijantimicag.2019.05.007.

Huang, Q.S., Liao, W., Xiong, Z., Li, D., Du, F.L., Xiang, T.X., et al. (2021). Prevalence of the NTEKPC-I on IncF Plasmids Among Hypervirulent Klebsiella pneumoniae Isolates in Jiangxi Province, South China. *Front Microbiol* 12**,** 622280. doi: 10.3389/fmicb.2021.622280.

Huang, Y.H., Chou, S.H., Liang, S.W., Ni, C.E., Lin, Y.T., Huang, Y.W., et al. (2018). Emergence of an XDR and carbapenemase-producing hypervirulent Klebsiella pneumoniae strain in Taiwan. *J Antimicrob Chemother* 73(8)**,** 2039-2046. doi: 10.1093/jac/dky164.

Jin, L., Liu, Y., Jing, C., Wang, R., Wang, Q., and Wang, H. (2020). Neutrophil extracellular traps (NETs)-mediated killing of carbapenem-resistant hypervirulent Klebsiella pneumoniae (CR-hvKP) are impaired in patients with diabetes mellitus. *Virulence* 11(1)**,** 1122-1130. doi: 10.1080/21505594.2020.1809325.

Jin, L., Wang, R., Gao, H., Wang, Q., and Wang, H. (2021). Identification of a Novel Hybrid Plasmid Encoding KPC-2 and Virulence Factors in Klebsiella pneumoniae Sequence Type 11. *Antimicrob Agents Chemother* 65(6). doi: 10.1128/aac.02435-20.

Karlsson, M., Stanton, R.A., Ansari, U., McAllister, G., Chan, M.Y., Sula, E., et al. (2019). Identification of a Carbapenemase-Producing Hypervirulent Klebsiella pneumoniae Isolate in the United States. *Antimicrob Agents Chemother* 63(7). doi: 10.1128/aac.00519-19.

Krapp, F., Morris, A.R., Ozer, E.A., and Hauser, A.R. (2017). Virulence Characteristics of Carbapenem-Resistant Klebsiella pneumoniae Strains from Patients with Necrotizing Skin and Soft Tissue Infections. *Sci Rep* 7(1)**,** 13533. doi: 10.1038/s41598-017-13524-8.

Lev, A.I., Astashkin, E.I., Kislichkina, A.A., Solovieva, E.V., Kombarova, T.I., Korobova, O.V., et al. (2018). Comparative analysis of Klebsiella pneumoniae strains isolated in 2012-2016 that differ by antibiotic resistance genes and virulence genes profiles. *Pathog Glob Health* 112(3)**,** 142-151. doi: 10.1080/20477724.2018.1460949.

Li, C., Ma, G., Yang, T., Wen, X., Qin, C., Yue, L., et al. (2020a). A rare carbapenem-resistant hypervirulent K1/ST1265 Klebsiella pneumoniae with an untypeable blaKPC-harboured conjugative plasmid. *J Glob Antimicrob Resist* 22**,** 426-433. doi: 10.1016/j.jgar.2020.04.009.

Li, D., Liao, W., Huang, H.H., Du, F.L., Wei, D.D., Mei, Y.F., et al. (2020b). Emergence of Hypervirulent Ceftazidime/Avibactam-Resistant Klebsiella pneumoniae Isolates in a Chinese Tertiary Hospital. *Infect Drug Resist* 13**,** 2673-2680. doi: 10.2147/idr.S257477.

Li, J., Huang, Z.Y., Yu, T., Tao, X.Y., Hu, Y.M., Wang, H.C., et al. (2019). Isolation and characterization of a sequence type 25 carbapenem-resistant hypervirulent Klebsiella pneumoniae from the mid-south region of China. *BMC Microbiol* 19(1)**,** 219. doi: 10.1186/s12866-019-1593-5.

Li, Y., Dong, L., Gao, W., Zhen, J., Dong, F., and Yao, K. (2021a). Hypervirulent Klebsiella pneumoniae Infections in Pediatric Populations in Beijing (2017-2019): Clinical Characteristics, Molecular Epidemiology and Antimicrobial Susceptibility. *Pediatr Infect Dis J* 40(12)**,** 1059-1063. doi: 10.1097/INF.0000000000003253.

Li, Y., Li, D., Xue, J., Ji, X., Shao, X., and Yan, J. (2021b). The Epidemiology, Virulence and Antimicrobial Resistance of Invasive Klebsiella pneumoniae at a Children's Medical Center in Eastern China. *Infect Drug Resist* 14**,** 3737-3752. doi: 10.2147/idr.S323353.

Liao, W., De Wang, L., Li, D., Du, F.L., Long, D., Liu, Y., et al. (2021). High Prevalence of 16s rRNA Methylase Genes Among Carbapenem-Resistant Hypervirulent Klebsiella pneumoniae Isolates in a Chinese Tertiary Hospital. *Microb Drug Resist* 27(1)**,** 44-52. doi: 10.1089/mdr.2019.0482.

Liu, B.T., and Su, W.Q. (2019). Whole genome sequencing of NDM-1-producing serotype K1 ST23 hypervirulent Klebsiella pneumoniae in China. *J Med Microbiol* 68(6)**,** 866-873. doi: 10.1099/jmm.0.000996.

Liu, C., Du, P., Zhao, J., Li, B., Wang, C., Sun, L., et al. (2020). Phenotypic and Genomic Characterization of Virulence Heterogeneity in Multidrug-Resistant ST11 Klebsiella pneumoniae During Inter-Host Transmission and Evolution. *Infect Drug Resist* 13**,** 1713-1721. doi: 10.2147/idr.S243836.

Liu, P.P., Liu, Y., Wang, L.H., Wei, D.D., and Wan, L.G. (2016). Draft Genome Sequence of an NDM-5-Producing Klebsiella pneumoniae Sequence Type 14 Strain of Serotype K2. *Genome Announc* 4(2). doi: 10.1128/genomeA.01610-15.

Liu, Y., Liu, P.P., Wang, L.H., Wei, D.D., Wan, L.G., and Zhang, W. (2017). Capsular Polysaccharide Types and Virulence-Related Traits of Epidemic KPC-Producing Klebsiella pneumoniae Isolates in a Chinese University Hospital. *Microb Drug Resist* 23(7)**,** 901-907. doi: 10.1089/mdr.2016.0222.

Liu, Z., Chu, W., Li, X., Tang, W., Ye, J., Zhou, Q., et al. (2021). Genomic Features and Virulence Characteristics of a Community-Acquired Bloodstream Infection-Causing Hypervirulent Klebsiella pneumoniae ST86 Strain Harboring KPC-2-Encoding IncX6 Plasmid. *Microb Drug Resist* 27(3)**,** 360-368. doi: 10.1089/mdr.2019.0394.

Liu, Z., Gu, Y., Li, X., Liu, Y., Ye, Y., Guan, S., et al. (2019). Identification and Characterization of NDM-1-producing Hypervirulent (Hypermucoviscous) Klebsiella pneumoniae in China. *Ann Lab Med* 39(2)**,** 167-175. doi: 10.3343/alm.2019.39.2.167.

Lorenzin, G., Gona, F., Battaglia, S., Spitaleri, A., Saluzzo, F., Trovato, A., et al. (2022). Detection of NDM-1/5 and OXA-48 co-producing extensively drug-resistant hypervirulent Klebsiella pneumoniae in Northern Italy. *J Glob Antimicrob Resist* 28**,** 146-150. doi: 10.1016/j.jgar.2022.01.001.

Mataseje, L.F., Boyd, D.A., Mulvey, M.R., and Longtin, Y. (2019). Two Hypervirulent Klebsiella pneumoniae Isolates Producing a bla (KPC-2) Carbapenemase from a Canadian Patient. *Antimicrob Agents Chemother* 63(7). doi: 10.1128/aac.00517-19.

Mendes, G., Ramalho, J.F., Bruschy-Fonseca, A., Lito, L., Duarte, A., Melo-Cristino, J., et al. (2022). First Description of Ceftazidime/Avibactam Resistance in a ST13 KPC-70-Producing Klebsiella pneumoniae Strain from Portugal. *Antibiotics (Basel)* 11(2). doi: 10.3390/antibiotics11020167.

Mohammad Ali Tabrizi, A., Badmasti, F., Shahcheraghi, F., and Azizi, O. (2018). Outbreak of hypervirulent Klebsiella pneumoniae harbouring bla(VIM-2) among mechanically-ventilated drug-poisoning patients with high mortality rate in Iran. *J Glob Antimicrob Resist* 15**,** 93-98. doi: 10.1016/j.jgar.2018.06.020.

Mukherjee, S., Naha, S., Bhadury, P., Saha, B., Dutta, M., Dutta, S., et al. (2020). Emergence of OXA-232-producing hypervirulent Klebsiella pneumoniae ST23 causing neonatal sepsis. *J Antimicrob Chemother* 75(7)**,** 2004-2006. doi: 10.1093/jac/dkaa080.

Octavia, S., Kalisvar, M., Venkatachalam, I., Ng, O.T., Xu, W., Sridatta, P.S.R., et al. (2019). Klebsiella pneumoniae and Klebsiella quasipneumoniae define the population structure of blaKPC-2Klebsiella: a 5 year retrospective genomic study in Singapore. *J Antimicrob Chemother* 74(11)**,** 3205-3210. doi: 10.1093/jac/dkz332.

Pajand, O., Darabi, N., Arab, M., Ghorbani, R., Bameri, Z., Ebrahimi, A., et al. (2020). The emergence of the hypervirulent Klebsiella pneumoniae (hvKp) strains among circulating clonal complex 147 (CC147) harbouring bla(NDM/OXA-48) carbapenemases in a tertiary care center of Iran. *Ann Clin Microbiol Antimicrob* 19(1)**,** 12. doi: 10.1186/s12941-020-00349-z.

Roulston, K.J., Bharucha, T., Turton, J.F., Hopkins, K.L., and Mack, D.J.F. (2018). A case of NDM-carbapenemase-producing hypervirulent Klebsiella pneumoniae sequence type 23 from the UK. *JMM Case Rep* 5(9)**,** e005130. doi: 10.1099/jmmcr.0.005130.

Saavedra, S.Y., Bernal, J.F., Montilla-Escudero, E., Arevalo, S.A., Prada, D.A., Valencia, M.F., et al. (2021). Complexity of Genomic Epidemiology of Carbapenem-Resistant Klebsiella pneumoniae Isolates in Colombia Urges the Reinforcement of Whole Genome Sequencing-Based Surveillance Programs. *Clin Infect Dis* 73(Suppl_4)**,** S290-S299. doi: 10.1093/cid/ciab777.

Sanikhani, R., Moeinirad, M., Solgi, H., Hadadi, A., Shahcheraghi, F., and Badmasti, F. (2021). The face of hypervirulent Klebsiella pneumoniae isolated from clinical samples of two Iranian teaching hospitals. *Ann Clin Microbiol Antimicrob* 20(1)**,** 58. doi: 10.1186/s12941-021-00467-2.

Scaltriti, E., Piccinelli, G., Corbellini, S., Caruso, A., Latronico, N., and De Francesco, M.A. (2020). Detection of a hypermucoviscous Klebsiella pneumoniae co-producing NDM-5 and OXA-48 carbapenemases with sequence type 383, Brescia, Italy. *Int J Antimicrob Agents* 56(4)**,** 106130. doi: 10.1016/j.ijantimicag.2020.106130.

Shaidullina, E., Shelenkov, A., Yanushevich, Y., Mikhaylova, Y., Shagin, D., Alexandrova, I., et al. (2020). Antimicrobial Resistance and Genomic Characterization of OXA-48- and CTX-M-15-Co-Producing Hypervirulent Klebsiella pneumoniae ST23 Recovered from Nosocomial Outbreak. *Antibiotics (Basel)* 9(12). doi: 10.3390/antibiotics9120862.

Shankar, C., Nabarro, L.E., Devanga Ragupathi, N.K., Muthuirulandi Sethuvel, D.P., Daniel, J.L., Doss, C.G., et al. (2016). Draft Genome Sequences of Three Hypervirulent Carbapenem-Resistant Klebsiella pneumoniae Isolates from Bacteremia. *Genome Announc* 4(6). doi: 10.1128/genomeA.01081-16.

Shao, C., Jin, Y., Wang, W., Jiang, M., and Zhao, S. (2021). An Outbreak of Carbapenem-Resistant Klebsiella pneumoniae of K57 Capsular Serotype in an Emergency Intensive Care Unit of a Teaching Hospital in China. *Front Public Health* 9**,** 724212. doi: 10.3389/fpubh.2021.724212.

Shen, Z., Gao, Q., Qin, J., Liu, Y., and Li, M. (2019). Emergence of an NDM-5-Producing Hypervirulent Klebsiella pneumoniae Sequence Type 35 Strain with Chromosomal Integration of an Integrative and Conjugative Element, ICEKp1. *Antimicrob Agents Chemother* 64(1). doi: 10.1128/aac.01675-19.

Shu, L., Dong, N., Lu, J., Zheng, Z., Hu, J., Zeng, W., et al. (2019). Emergence of OXA-232 Carbapenemase-Producing Klebsiella pneumoniae That Carries a pLVPK-Like Virulence Plasmid among Elderly Patients in China. *Antimicrob Agents Chemother* 63(3). doi: 10.1128/aac.02246-18.

Solgi, H., Shahcheraghi, F., Bolourchi, N., and Ahmadi, A. (2020). Molecular characterization of carbapenem-resistant serotype K1 hypervirulent Klebsiella pneumoniae ST11 harbouring bla(NDM-1) and bla(OXA-48) carbapenemases in Iran. *Microb Pathog* 149**,** 104507. doi: 10.1016/j.micpath.2020.104507.

Starkova, P., Lazareva, I., Avdeeva, A., Sulian, O., Likholetova, D., Ageevets, V., et al. (2021). Emergence of Hybrid Resistance and Virulence Plasmids Harboring New Delhi Metallo-β-Lactamase in Klebsiella pneumoniae in Russia. *Antibiotics (Basel)* 10(6). doi: 10.3390/antibiotics10060691.

Su, C., Wu, T., Meng, B., Yue, C., Sun, Y., He, L., et al. (2021). High Prevalence of Klebsiella pneumoniae Infections in AnHui Province: Clinical Characteristic and Antimicrobial Resistance. *Infect Drug Resist* 14**,** 5069-5078. doi: 10.2147/idr.S336451.

Su, S., Zhang, J., Zhao, Y., Yu, L., Wang, Y., Wang, Y., et al. (2020). Outbreak of KPC-2 Carbapenem-resistant Klebsiella pneumoniae ST76 and Carbapenem-resistant K2 Hypervirulent Klebsiella pneumoniae ST375 strains in Northeast China: molecular and virulent characteristics. *BMC Infect Dis* 20(1)**,** 472. doi: 10.1186/s12879-020-05143-y.

Turton, J.F., Payne, Z., Coward, A., Hopkins, K.L., Turton, J.A., Doumith, M., et al. (2018). Virulence genes in isolates of Klebsiella pneumoniae from the UK during 2016, including among carbapenemase gene-positive hypervirulent K1-ST23 and 'non-hypervirulent' types ST147, ST15 and ST383. *J Med Microbiol* 67(1)**,** 118-128. doi: 10.1099/jmm.0.000653.

Wei, D.D., Wan, L.G., and Liu, Y. (2018). Draft Genome Sequence of an NDM-1- and KPC-2-Coproducing Hypervirulent Carbapenem-Resistant Klebsiella pneumoniae Strain Isolated from Burn Wound Infections. *Genome Announc* 6(13). doi: 10.1128/genomeA.00192-18.

Wei, T., Zou, C., Qin, J., Tao, J., Yan, L., Wang, J., et al. (2022). Emergence of Hypervirulent ST11-K64 Klebsiella pneumoniae Poses a Serious Clinical Threat in Older Patients. *Front Public Health* 10**,** 765624. doi: 10.3389/fpubh.2022.765624.

Xie, M., Yang, X., Xu, Q., Ye, L., Chen, K., Zheng, Z., et al. (2021). Clinical evolution of ST11 carbapenem resistant and hypervirulent Klebsiella pneumoniae. *Commun Biol* 4(1)**,** 650. doi: 10.1038/s42003-021-02148-4.

Xu, M., Fu, Y., Fang, Y., Xu, H., Kong, H., Liu, Y., et al. (2019). High prevalence of KPC-2-producing hypervirulent Klebsiella pneumoniae causing meningitis in Eastern China. *Infect Drug Resist* 12**,** 641-653. doi: 10.2147/idr.S191892.

Yan, R., Lu, Y., Zhu, Y., Lan, P., Jiang, S., Lu, J., et al. (2021). A Sequence Type 23 Hypervirulent Klebsiella pneumoniae Strain Presenting Carbapenem Resistance by Acquiring an IncP1 bla (KPC-2) Plasmid. *Front Cell Infect Microbiol* 11**,** 641830. doi: 10.3389/fcimb.2021.641830.

Yang, Q., Jia, X., Zhou, M., Zhang, H., Yang, W., Kudinha, T., et al. (2020). Emergence of ST11-K47 and ST11-K64 hypervirulent carbapenem-resistant Klebsiella pneumoniae in bacterial liver abscesses from China: a molecular, biological, and epidemiological study. *Emerg Microbes Infect* 9(1)**,** 320-331. doi: 10.1080/22221751.2020.1721334.

Yang, X., Dong, N., Liu, X., Yang, C., Ye, L., Chan, E.W., et al. (2021). Co-conjugation of Virulence Plasmid and KPC Plasmid in a Clinical Klebsiella pneumoniae Strain. *Front Microbiol* 12**,** 739461. doi: 10.3389/fmicb.2021.739461.

Yang, X., Sun, Q., Li, J., Jiang, Y., Li, Y., Lin, J., et al. (2022a). Molecular epidemiology of carbapenem-resistant hypervirulent Klebsiella pneumoniae in China. *Emerg Microbes Infect* 11(1)**,** 841-849. doi: 10.1080/22221751.2022.2049458.

Yang, Y., Yang, Y., Ahmed, M., Qin, M., He, R., Wu, Y., et al. (2022b). Carriage of distinct bla(KPC-2) and bla(OXA-48) plasmids in a single ST11 hypervirulent Klebsiella pneumoniae isolate in Egypt. *BMC Genomics* 23(1)**,** 20. doi: 10.1186/s12864-021-08214-9.

Yao, B., Xiao, X., Wang, F., Zhou, L., Zhang, X., and Zhang, J. (2015). Clinical and molecular characteristics of multi-clone carbapenem-resistant hypervirulent (hypermucoviscous) Klebsiella pneumoniae isolates in a tertiary hospital in Beijing, China. *Int J Infect Dis* 37**,** 107-112. doi: 10.1016/j.ijid.2015.06.023.

Yonekawa, S., Mizuno, T., Nakano, R., Nakano, A., Suzuki, Y., Asada, T., et al. (2020). Molecular and Epidemiological Characteristics of Carbapenemase-Producing Klebsiella pneumoniae Clinical Isolates in Japan. *mSphere* 5(5). doi: 10.1128/mSphere.00490-20.

Yuan, Y., Li, Y., Wang, G., Li, C., Chang, Y.F., Chen, W., et al. (2019). bla (NDM-5) carried by a hypervirulent Klebsiella pneumoniae with sequence type 29. *Antimicrob Resist Infect Control* 8**,** 140. doi: 10.1186/s13756-019-0596-1.

Zhan, L., Wang, S., Guo, Y., Jin, Y., Duan, J., Hao, Z., et al. (2017). Outbreak by Hypermucoviscous Klebsiella pneumoniae ST11 Isolates with Carbapenem Resistance in a Tertiary Hospital in China. *Front Cell Infect Microbiol* 7**,** 182. doi: 10.3389/fcimb.2017.00182.

Zhang, R., Lin, D., Chan, E.W., Gu, D., Chen, G.X., and Chen, S. (2016). Emergence of Carbapenem-Resistant Serotype K1 Hypervirulent Klebsiella pneumoniae Strains in China. *Antimicrob Agents Chemother* 60(1)**,** 709-711. doi: 10.1128/aac.02173-15.

Zhang, Y., Jin, L., Ouyang, P., Wang, Q., Wang, R., Wang, J., et al. (2020). Evolution of hypervirulence in carbapenem-resistant Klebsiella pneumoniae in China: a multicentre, molecular epidemiological analysis. *J Antimicrob Chemother* 75(2)**,** 327-336. doi: 10.1093/jac/dkz446.

Zhang, Y., Wang, X., Wang, S., Sun, S., Li, H., Chen, H., et al. (2021). Emergence of Colistin Resistance in Carbapenem-Resistant Hypervirulent Klebsiella pneumoniae Under the Pressure of Tigecycline. *Front Microbiol* 12**,** 756580. doi: 10.3389/fmicb.2021.756580.

Zhang, Y., Zeng, J., Liu, W., Zhao, F., Hu, Z., Zhao, C., et al. (2015). Emergence of a hypervirulent carbapenem-resistant Klebsiella pneumoniae isolate from clinical infections in China. *J Infect* 71(5)**,** 553-560. doi: 10.1016/j.jinf.2015.07.010.

Zhao, J., Zhang, Y., Fan, Y., Han, J., Xiong, Z., Liu, X., et al. (2021). Characterization of an NDM-5-producing hypervirulent Klebsiella pneumoniae sequence type 65 clone from a lung transplant recipient. *Emerg Microbes Infect* 10(1)**,** 396-399. doi: 10.1080/22221751.2021.1889932.

Zheng, B., Xu, H., Lv, T., Guo, L., Xiao, Y., Huang, C., et al. (2020). Stool Samples of Acute Diarrhea Inpatients as a Reservoir of ST11 Hypervirulent KPC-2-Producing Klebsiella pneumoniae. *mSystems* 5(3). doi: 10.1128/mSystems.00498-20.

Zhou, C., Wu, Q., He, L., Zhang, H., Xu, M., Yuan, B., et al. (2021). Clinical and Molecular Characteristics of Carbapenem-Resistant Hypervirulent Klebsiella pneumoniae Isolates in a Tertiary Hospital in Shanghai, China. *Infect Drug Resist* 14**,** 2697-2706. doi: 10.2147/idr.S321704.

Zhou, K., Xiao, T., David, S., Wang, Q., Zhou, Y., Guo, L., et al. (2020). Novel Subclone of Carbapenem-Resistant Klebsiella pneumoniae Sequence Type 11 with Enhanced Virulence and Transmissibility, China. *Emerg Infect Dis* 26(2)**,** 289-297. doi: 10.3201/eid2602.190594.

Zhou, S., Ren, G., Liu, Y., Liu, X., Zhang, L., Xu, S., et al. (2022). Challenge of evolving Klebsiella pneumoniae infection in patients on hemodialysis: from the classic strain to the carbapenem-resistant hypervirulent one. *Int J Med Sci* 19(3)**,** 416-424. doi: 10.7150/ijms.69577.

Zhou, Y., Wang, X., Shen, J., Lu, Z., and Liu, Y. (2019). Endogenous Endophthalmitis Caused by Carbapenem-Resistant Hypervirulent Klebsiella Pneumoniae: A Case Report and Literature Review. *Ocul Immunol Inflamm* 27(7)**,** 1099-1104. doi: 10.1080/09273948.2018.1502786.
